# The importance of patch shape at threshold occupancy: functional patch size within total habitat amount

**DOI:** 10.1007/s00442-023-05453-3

**Published:** 2023-10-10

**Authors:** Jeffrey K. Keller, Patrick J. Sullivan

**Affiliations:** 1 Department of Natural Resources and the Environment, Cornell University, Ithaca, NY 14853 USA; 2 Present Address: Habitat by Design, 74 Stagecoach Road, Pipersville, PA 18947 USA

**Keywords:** Species richness, Habitat amount hypothesis, Landscape metrics, Spatial resolution, Guild

## Abstract

The habitat amount hypothesis (HAH) stresses the importance of total patch amount over the size of individual patches in determining species richness within a local landscape. However, the absence of some species from patches too small to contain a territory would be inconsistent with the HAH. Using the association of territory size with body size and the circle as optimal territory shape, we tested several HAH predictions of threshold patch occupancy and richness of 19 guilds of primarily insectivorous breeding birds. We characterized 16 guild-associated patch types at high spatial resolution and assigned one type to each guild. We measured functional patch size as the largest circle that fit within each patch type occurring in a local landscape. Functional patch size was the sole or primary predictor in regression models of species richness for 15 of the 19 guilds. Total patch amount was the sole or primary variable in only 2 models. Quantifying patch size at high resolution also demonstrated that breeding birds should be absent from patches that are too small to contain a territory and larger species should occur only in larger patches. Functional patch size is a readily interpretable metric that helps explain the habitat basis for differences in species composition and richness between areas. It provides a tool to assess the combined effects of patch size, shape and perforation on threshold habitat availability, and with total patch amount can inform design and/or evaluation of conservation, restoration or enhancement options for focal taxa or biodiversity in general.

**Supplementary Information:**

The online version contains supplementary material available at 10.1007/s00442-023-05453-3.

## Introduction

The habitat amount hypothesis (HAH) posits that within a local landscape, species richness in a given patch type, termed the “habitat patch”, is simply a function of the total habitat amount in the landscape, regardless of the sizes of the individual habitat patches in that landscape (Fahrig 2013). Although the hypothesis has generated debate, mostly about its mechanisms and consequences (e.g., Hanski 2015; Fletcher et al. 2018; Deane 2022), it has garnered considerable empirical support (reviewed by Watling et al. 2020). Additionally, few analyses of the HAH to date have found evidence for an effect of patch size alone on richness or occurrence, a finding that would be inconsistent with the HAH (e.g., Haddad et al. 2017; MacDonald et al. 2021).

**Fig. 1:**
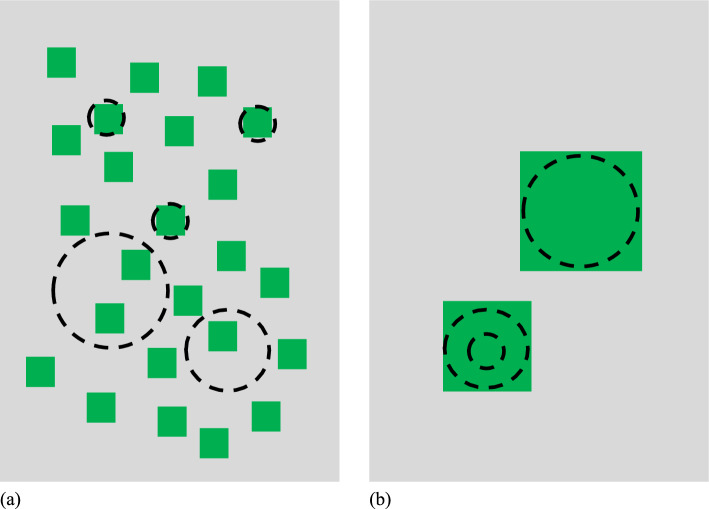
Two hypothetical local landscapes with identical total amounts of a patch type (green boxes) suitable for 3 species differing in territory size requirements (dashed black circles). If all patches within (**a**) are only large enough for occupancy by the species with the smallest territory (smallest circles) but territories for all 3 species could be accommodated in the larger patches in (**b**), this would indicate an effect of patch size on species richness beyond the sample area effect (total patch amount)

One possible expression of the influence of patch size alone on species richness not considered by the HAH relates to the difference in territory sizes among species that defend all-purpose territories (nesting, resting, breeding, feeding) (Fahrig 2013, p. 1655; Deane 2022). Among such species, patch occupancy depends on the presence of a sufficiently sized single patch of habitat (i.e., scale dependence) to contain a territory (Keller 1986; Hinsley et al. 1996; Lindenmayer et al. 1999; Beier et al. 2002). Thus, species with territories larger than all available patches should be absent from the local landscape (Fig. 1; compare with Watling et al. 2020, fig 1c and d) and patch size should be an important predictor not only of threshold occupancy for individual species but of richness. This would indicate an effect of patch size beyond the sample area effect, the basis of the HAH.

**Fig. 2:**
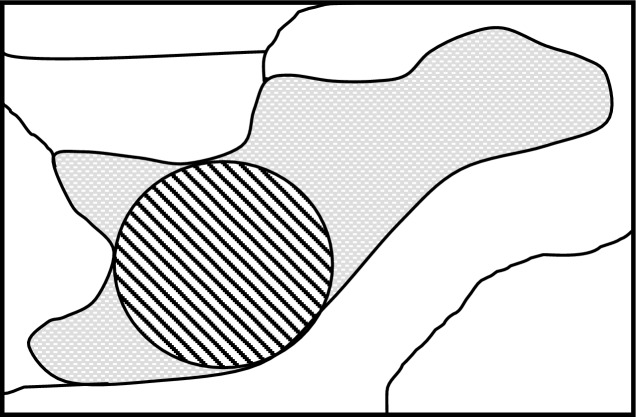
The Maximum Diameter Circle (MDC), representing the largest optimally shaped (i.e., most energetically efficient) territory that fits within a “solid” patch type (e.g., deciduous forest) located within other cover types identifiable on remotely sensed imagery. MDC represents the functional size of the patch. (From Keller 1986 and Keller and Smith 2014)

**Fig. 3:**
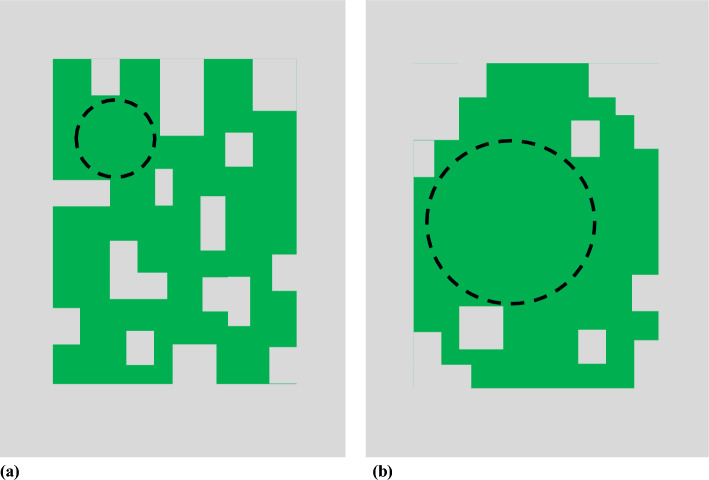
Two solid patches of identical type and amount illustrating the potential influence on threshold occupancy of different levels of perforation by non-patch landscape component types. The less perforated arrangement at right (**b**) supports a 2 × larger diameter (4 × larger area) MDC (dashed circle) than in (**a**). MDC represents the functional size of the patch (i.e., largest, most energetically efficient configuration) for territory establishment

However, because patches are not shaped uniformly and may be perforated, patch size per se does not automatically equate to maximum potential territory size. This is because all-purpose territories tend to be circular, both theoretically (Covich 1976) and empirically (Grant 1968; fig. 1, Moorcroft et al. 1999) due to the energetics of territory defense (minimum perimeter to area ratio) and optimal foraging efficiency. As a result, patches of the same size but with differing shapes (i.e., perimeter to area ratios (Fig. 2)) or degrees of internal perforation (Fig. 3), may differ in their ability to support a particular species. Under these commonly occurring conditions, threshold patch size for territorial species may be better approximated by determining the size of the largest circle (optimally shaped territory) that fits within the patch of interest (Fig. 2, Keller and Smith 2014). This maximum diameter circle (MDC) represents the functional size of the patch.

**Fig. 4:**
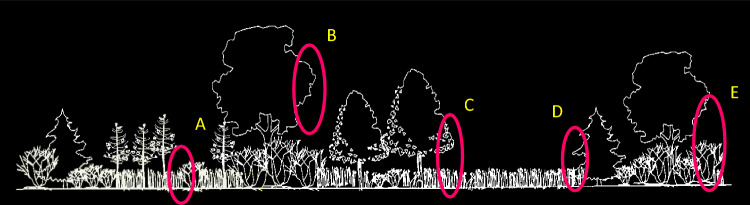
A perspective view of a mid-stage successional oldfield illustrating 5 different types of edge to which various species of wildlife respond. **A** shrub/grass, **B** deciduous canopy/open air, **C** deciduous sapling-poletimber/grass, **D** coniferous sapling-poletimber/grass, **E** deciduous canopy/deciduous shrub. Although each of these edge types has meaning to different species of wildlife, edges viewed at this scale often are not considered in analyses of species richness or in conservation planning. (From Keller and Smith 2014)

Several guidelines have been offered to properly explore the tenets of the HAH (Fahrig 2013, p. 1656). First, “habitat” (constituting a patch), whether contiguous or edge (Fahrig 2013, fig. 8), should be correctly defined for the species or species group included in the species richness estimate. This suggests identifying more restrictively defined species subgroups associated with more homogeneous patch types (MacDonald et al. 2021). This reduces the complicating influence on species richness of increasing environmental heterogeneity inherent in more broadly defined plant communities or other biotopes (Wiens 1989; Ricklefs and Lovette 1999; Tews et al. 2004). For example, an assemblage of shrubland birds associated with a broad cover type such as early successional forest (e.g., clearcuts, oldfields) would likely include multiple species groups (guilds). Each group would be associated with a vegetation subset of such forests, with members linked behaviorally by natural history traits such as foraging attributes and nest placement. Guild-associated vegetation subsets would include contiguous patch types (Fahrig 2013, Fig. 8) such as dense shrubs used by low (0–3 m) foliage-gleaning insectivorous birds and edge types such as shrubs adjacent to open grass (Fig. 4) used by terrestrial gleaners (Keller et al. 2003).

Second, analysis should be at the spatial scale with the strongest species richness-habitat amount relationship (i.e., the scale of effect (Jackson and Fahrig 2012)). This scale is likely related to average movement ranges of species in a group, which is itself often related to territory size, both of which are frequently functions of body size (Schoener 1968; Peters 1983; Kelt and Varen 2001; Perry and Garland 2002). For GIS-based analyses of assemblages that include smaller species that use landscapes in a more fine-grained way than larger species (Cushman et al. 2008), appropriate image spatial resolution is at least as important as appropriate scale to avoid missing critical species-habitat relationships. This is true regardless of how many scales of analysis are investigated (Keller and Smith 2014).

Despite the precise use of the term habitat as being species specific (Mathewson and Morrison 2015), it has often been used in a more general sense, such as “forest habitat.” Patch, too, has been variously defined, first by MacArthur et al. (1962) as a species-associated foliage profile, and later by Wiens (1976) as “a surface area differing from its surroundings in nature and appearance”. In GIS, patches are the polygons (vector-based) or cell clusters (raster-based) of the GIS map. Keller and Smith (2014, pp. 12–15) argued that edge too, is consistent with Wiens (1976) definition of patch as representing an area because the historic value of edge always has been considered to lie in the composition of its adjacent components (e.g., one component used for foraging, the other used for nesting and escape cover, Fig. 4), not simply the linear interface between them (Leopold 1933). As a result, they suggested definitions for two general patch (as area) associations of wildlife separable in a GIS landscape image–“solid” and “edge” (Keller 1986; Keller et al. 2003; Keller and Smith 2014). Solid patches are clusters of identical or structurally similar landscape components (e.g., open grass, open water, sawtimber trees [i.e., a forest], emergent marsh) associated with a particular species or assemblage. Edge patches are any combination of interfaces between adjacent structurally dissimilar landscape components (e.g., shrub-grass, deciduous tree-grass, open water-emergent marsh) associated with a particular species or assemblage. Keller and Smith (2014) also argued that per Risser (1987) and Wiens (1989) edge habitat is not limited to edge at the scale of ecotones (Online Resource [OR] 1; Clements 1905; Odum 1971; Ries et al. 2004) as most often considered in GIS-based studies but can occur at any scale and resolution (ecological grain) (Fig. 4 and OR 2).

Applying the concept of solid and edge patch types allows partitioning of a species assemblage into subgroups of functionally similar species based on shared natural history traits such as foraging heights, foraging tactics and nest heights. Each subgroup has an association with a definable subset (patch type) of the various landscape components found across the cover types studied. Thus, as defined here, “patch” can be considered the habitat of a guild (Keller and Smith 1983; Keller 1986; Keller et al. 2003).

Used in conjunction with high resolution (HR) remotely sensed imagery and examining patch attributes at the territory scale of passerines, the patch-as-guild-habitat approach has several potential advantages over lower resolution (e.g., Landsat-based) analyses of more generalized cover types (e.g., forest vs. nonforest) for evaluating aspects of the HAH and applying the results to conservation or management. First, the use of HR imagery fosters identification of a suite of covariates based on organism-centric patch types that allow for more species-scaled delineation of patch boundaries (Kotliar and Wiens 1990). Second, beyond highlighting differences in species richness between one area and another, this approach aids in understanding differences in species composition due to spatial attributes and differential availability of guild-specific patch types (Keller and Smith 2014, Chap. 6). Lastly, it is useful in designing and evaluating the effects of conservation or management options to preserve, enhance or create habitat for individual species or promote biodiversity in general (Keller 2021).

Using a pre-existing dataset, guilds as our species groups, and measures of functional patch size and total patch amount of guild-specific solid and edge patch types, we tested the following predictions of species richness and threshold occurrence for each guild, including several discussed by Fahrig (2013, p. 1658). Support for a prediction would be inconsistent with the HAH:

- Intraguild species richness is better predicted by the functional size of guild-specific patches than it is by total patch amount;
- A guild will be absent in the local landscape unless at least one patch of the type specific to the guild is functionally large enough (i.e., threshold size) to support a territory, regardless of total patch amount;
- In multispecies guilds, given the relationship of territory size to body size, if only one species is present, it should be the smallest species;
- In multispecies guilds, larger species occur only in functionally larger patches;
- Intraguild richness increases with the functional size of guild-specific patches, not necessarily with size of the general cover/plant community type in which the patch occurs (Holland et al. 2005).

## Methods

The study was conducted at the 4850 ha Connecticut Hill Wildlife Management Area (Connecticut Hill WMA) in Tompkins County, New York (N. Lat. 43° 21.8′, W Long. 76° 40.7′) (Keller et al. 2003). Twenty three clearcuts, oldfields, and forests ranging in age from 2 to 120 years old and in size from 1.0 to 24 ha were studied during the five-year period 1977–1981 (OR 3). Each site, equivalent to a local landscape for this analysis, was surveyed for breeding birds for 2 to 5 years (column 2, OR 3; locations in Appendix IV of Keller 1980) resulting in a total of 97 survey-years of data. All but the 4 oldest (55–120 years old) sites were discrete entities and were surveyed in their entirety. Surveys of forest interiors within 4 extensive older forest stands (55 to 120 year old) were conducted along a 180 m wide transect within each stand. Transect limits were located more than 100 m from forest edges (fig. 1, Keller 1986). We conducted breeding bird surveys each year between late May and mid-July using a modified form of the spot-map census technique (International Bird Census Committee 1970). Each site was surveyed 5–8 times during the breeding season, including extended supplemental late morning and early evening visits. Breeding season time spent on each site during the course of the study was proportional to site size with between 40 and 130 h of observer time devoted to bird surveys, sampling vegetation profiles and snag density (Keller et al. 2003), and collecting insect and small mammal samples (Keller 1986).

### Organizational level of analysis

In response to the caution to carefully define habitat for species groups of interest (Fahrig 2013), we use the term guild to describe subsets of the avifauna because it conveys the shared habitat characteristics and functional similarity of species designated as members of the same guild. Examining guild associations with patches defined as subsets of broader plant communities also allows for more direct comparison of the effects of functional patch size and total patch amount by reducing the influence on assemblage richness of the heterogeneity inherent in broadly defined cover types (Fletcher et al. 2018). The 59 species in the analysis (OR 4) were grouped into 19 guilds (Keller et al. 2003, OR 5), including 6 single-species guilds of habitat specialists. For ease of reference, guilds were numbered 1–19 (OR 6). Note, the choice to partition the total avian assemblage into smaller functional groups meant that maximum richness of the identified guilds was arguably small for testing predictions of species richness. However, we felt this was justified because the approach addressed both the increased within-patch homogeneity sought by Fahrig (2013, p. 1656) and a criticism that using multispecies analyses to test the HAH may confound species-specific habitat requirements (Hanski 2015; Torrenta and Villard 2017). Furthermore, 3 of our questions did not involve species richness, but focused instead on habitat availability (i.e., was there a patch size threshold for each guild?) or nonrandom species occurrence. Avian nomenclature throughout the text follows the American Ornithologists’ Union Check-list (Chesser et al. 2019).

### Vegetation mapping, scale of analysis and image resolution and classification

Most studies of species-landscape relationships have not included relevant scales of space use such as home range or territory size (Jackson and Fahrig 2015). In consideration of the territory size (scale) and inferred spatial resolution (grain) of habitat use by territorial, breeding passerines, we obtained HR aerial photography on 22-May-1977 and 10-Jun-1980 from an altitude of 1100 m using a Hasselblad camera and 70 mm black and white film.

The imagery, which had spatial resolution (ground resolvable distance [GRD]) to < 0.75 m (National Imagery Interpretability Rating Scale [NIIRS] Level 6 [Keller and Smith 2014, appendix A]), allowed identification of individual small shrubs and saplings. For each year of photographs, we used multiple stereo pairs of each site at a scale of 1:5000 to interpret and map vegetation on geo-rectified base maps produced at a scale of 1:2000 (OR 2) using a classification system of 16 landscape component types (OR 7). Component types and percent cover categories were selected based on traditional remote sensing classifications and an extensive literature review of species-habitat associations (see guild classification in the following section). Classification accuracy of landscape components was verified by ground surveys of all sites.

For both interpretability at the 1:2000 scale and biological appropriateness, we chose a map cell size (minimum mapping unit [MMU]) equivalent to 100 m^2^ on the ground (left-hand grid, single hexagonal cells, OR 2). Therefore, each cell classified individual, intraterritory structural elements that have been suggested as critical to habitat selection in birds (Hilden 1965; Cody 1981; Klopfer and Ganzhorn 1985). We transferred maps of component types (e.g., dense sprouts or shrubs, deciduous sawtimber, deciduous sapling-poletimber, open grass) to a hexagonal-celled GIS called SPADIST (OR 2, left-hand grid), developed to analyze the horizontal heterogeneity of young clearcuts and oldfields in the study (Keller et al. 1979a; Keller 1986). Advantages of hexagonal vs. square cells for edge quantification in GIS are discussed in Keller et al. (1979b).

### Patch type identification

To describe a suite of patch types (any one of which is referred to generally as Patch Type T) thought to be used by the identified guilds (OR 6), we identified combinations of landscape components from OR 7 that characterize subsets of the landscape as either (1) solid patch types such as dense shrubs (patch type 1, Table 1, composition detailed in OR 8) and open grasslands (patch type 7), or (2) edge patch types such as shrub-opening edge (patch type 9, Fig. 4A) and canopy-opening edge (patch type 11, Fig. 4B). We identified an exploratory (first cut) suite of 16 patch types (OR 8) and for the purpose of comparing the influence of total patch amount and functional patch size on species richness assigned only one patch type to each of the 19 avian guilds (Table 1) based on a literature review (e.g., Kendeigh 1945; Hespenheide 1971; Holmes et al. 1979; Hamel et al. 1982; Schlossberg et al. 2010), discussions with colleagues, and personal observations during 8 field seasons. The resulting assignment, which for ease of reference paired patch types 1–16 with Guilds 1–16, classified 9 guilds as associated with solid patch types and 10 guilds as associated with edge patch types (Table 1). Note that different patch types may have certain landscape components in common due to overlaps in space use by their associated guilds. For example, foliage gleaners (Guild/patch 9) and flycatchers (Guilds/patch types 10 and 11) may both have sapling size deciduous trees (component type 5, OR 8; Fig. 4C) within their territories but differ in other structural components. Patch specificity of guilds also meant that not all guilds occurred on all sites, which resulted in differing sample sizes between guilds for certain analyses.

**Table 1:** Working associations of 19 guilds of breeding birds with exploratory GIS-based patch types (T) at the Connecticut Hill WMA in central New York

Patch type (T)	Description	Associated Guild^b^
Number^a^		
1	Deciduous dense shrubs	1
2	Deciduous middle canopy	2
3	Deciduous high canopy	3, 17, 18
4	Open shrub (shrub mixed with grass)	4
5	Deciduous understory	5
6	Mixed deciduous coniferous canopy	6
7	Open grass	7
8	Coniferous/opening	8
9	Shrub/opening	9
10	Sapling/opening	10, 19
11	Canopy/opening	11
12	Canopy/shrub	12
13	Northern hardwood-hemlock/shrub	13
14	Shrub-sapling/opening	14
15	Mid-canopy/opening	15
16	Coniferous canopy/shrub	16

^a^Patch numbering system follows Keller 1986 and Keller and Smith 2014. Patch types 1–7 are solid types (see text). Patch types 8–16 (i.e., those whose description includes a ‘/’) are edge types. See OR 7 for the landscape component classification system and OR 8 for the combination of individual components composing each patch type

^b^Guild numbers refer to those in OR 6

Most prior tests of the HAH have defined species groups and habitat patch types broadly, equivalent to community-level assemblages and biotopes, respectively (Melo et al. 2017; Rabelo et al. 2017; Torrenta and Villard 2017; Vieira et al. 2018; Halstead et al. 2019). If analyzed similarly, the total assemblage considered here could be categorized into 3 main groups of breeding birds—open grassland species, shrubland species, and mid-stage to mature forest species. To compare the guild approach with the broader species group classification typically examined in tests of the HAH, we also grouped the more than 25 shrubland species (Guilds 1, 2, 4, 5, 8, 9, 10, 14, 15, 19) into a larger assemblage. Shrublands included oldfields and early successional forest (Sites CCA, CCB, CCC, and OF; N = 79, OR 3).

### Predictor variables

Patch size (of solid patches) is typically measured as total area within the perimeter of a patch, regardless of patch shape. However, Covich (1976) argued that in horizontal space a circle is the energetically optimal shape for an all-purpose territory, which suggests that patches of the same size but with different shapes (e.g., square, linear, amoebic) represent different functional sizes to potential colonizers (Fig. 2). Therefore, to locate the functionally largest patch of each type on the GIS maps, we measured the diameter (m) of the maximum-sized circle (Maximum Diameter Circle [MDC], Table 2) that fit within each solid patch type (1–7, OR 8), on each site directly from the 1:2000 hexagonal-celled 1977 and 1980 vegetation maps (OR 9).

**Table 2:** Predictor variables used in the analysis of avian guild-patch relationships

Variable	Description
DEAC (8–16^a^)	Diameter (m) of the equivalent area circle in an edge patch type (T). See Fig. 5 and OR 11
DEACA (8–16)	The area of the circle with diameter DEAC for patch type T
MDC (1–7)	Maximum diameter (m) circle in a solid patch type (T). See Fig. 2 and OR 9
MDCA (1–7)	The area of the circle with diameter MDC for patch type T
NUMHAB (1–16)	Number of landscape components^b^ of patch type T (OR 8) actually present on a site
SITESIZE	Size (ha) of each of the 23 bird survey sites
SNAGTOT	Total density (no. ha-2) of > 5 cm dbh snags on a site
TOTAMT (1–16)	Area (number of cells) of a solid patch type (T) or edge length (# of cell sides) of an edge patch type (T) on a site

^a^Numbers in parentheses refer to patch types (T) (Table 1)

^b^For edge patches, a component is one of the pairs of adjacent landscape elements that define a particular edge patch type (OR 8)

**Fig. 5:**
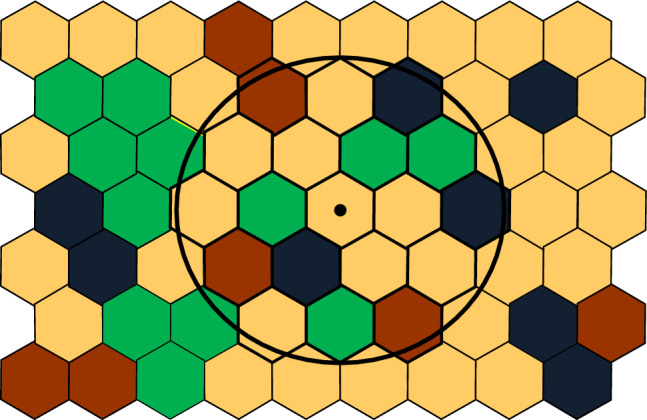
An example of the DEAC (Diameter of the Equivalent Area Circle) for shrub-sapling/opening edges (Patch Type 14, Table 1, OR 8) resulting from an ESCAN edge analysis of a hypothetical oldfield. Every cell on the map is the origin of a prespecified number of annular samples that records the number of patch type T edges within each sample size (OR 10). After locating the highest density (m m-2) on the map of type T edges for each sample size, the program compares edge density across all sample sizes (OR 11, column 1) and selects the sample size with the highest absolute edge density (OR 11, column “EI”). The program then converts the actual area sampled (AAS) for this EI value into an equivalently sized circle (DEAC) comparable (for edges) to solid patch type MDC (Fig. 2). Note, for illustrative purposes, all plot perimeter edge is non-type T (i.e., all type T is internal). In practice, edge between the plot and adjacent cover types also could contain type T edges. Black cells = coniferous trees, brown cells = deciduous trees, green cells = deciduous shrubs, tan cells = grass. (From Keller and Smith 2014)

To measure the size of edge patches in a manner comparable to MDC, Keller (1986) developed an edge-scanning algorithm (ESCAN). ESCAN searches a prespecified number of annular samples around every cell on the map (OR 10) and records the number of patch type T edges within each sample size (e.g., shrub-sapling/opening edge, Table 1, Type 14, Fig. 5). The program locates the area on the map with the highest density (m m-2) of type T edges for each sample size (i.e., number of annuli), then compares edge density across all sample sizes (OR 11, column 1) and selects the sample size with the highest absolute edge density (OR 11, column header EI). This sample size is then converted to the diameter of the equivalent area circle (DEAC) (Fig. 5; OR 11, last column). Because MDC and DEAC are based on incremental (i.e., single cell width) exploding scans across an entire local landscape, they are arguably surrogates for determining the scale of effect (Jackson and Fahrig 2012) and produce measures of functional patch size appropriate for exploring the HAH.

Total patch amount (TOTAMT) for solid patch types 1–7 was calculated as the area (number of all cells) of patch type T on each site for each survey year. TOTAMT for edge patch types 8–16 was the sum of the length of all edges (number of cell sides; cell side = 6.2 m) of patch type T (Fig. 5) on each site for each survey year.

As a related alternative to circle diameter (MDC and DEAC), we also calculated circle area (m^2^) (MDCA and DEACA, Table 2). Total density of snags (SNAGTOT) was calculated from snag data collected on all sites (Keller et al. 2003, p. 547). The size (SITESIZE, OR 3) and internal heterogeneity (NUMHAB) of each local landscape were derived from the GIS maps (Keller 1986).

### Data analysis

**Fig. 6:**
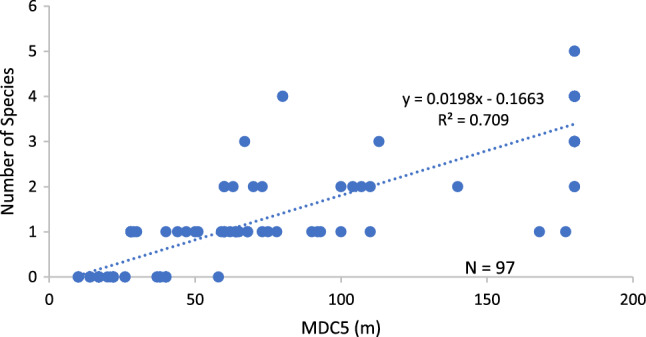
Linear regression model of Guild 5 (DUGI1) species richness vs. functional patch size (m). See Table 3

**Fig. 7:**
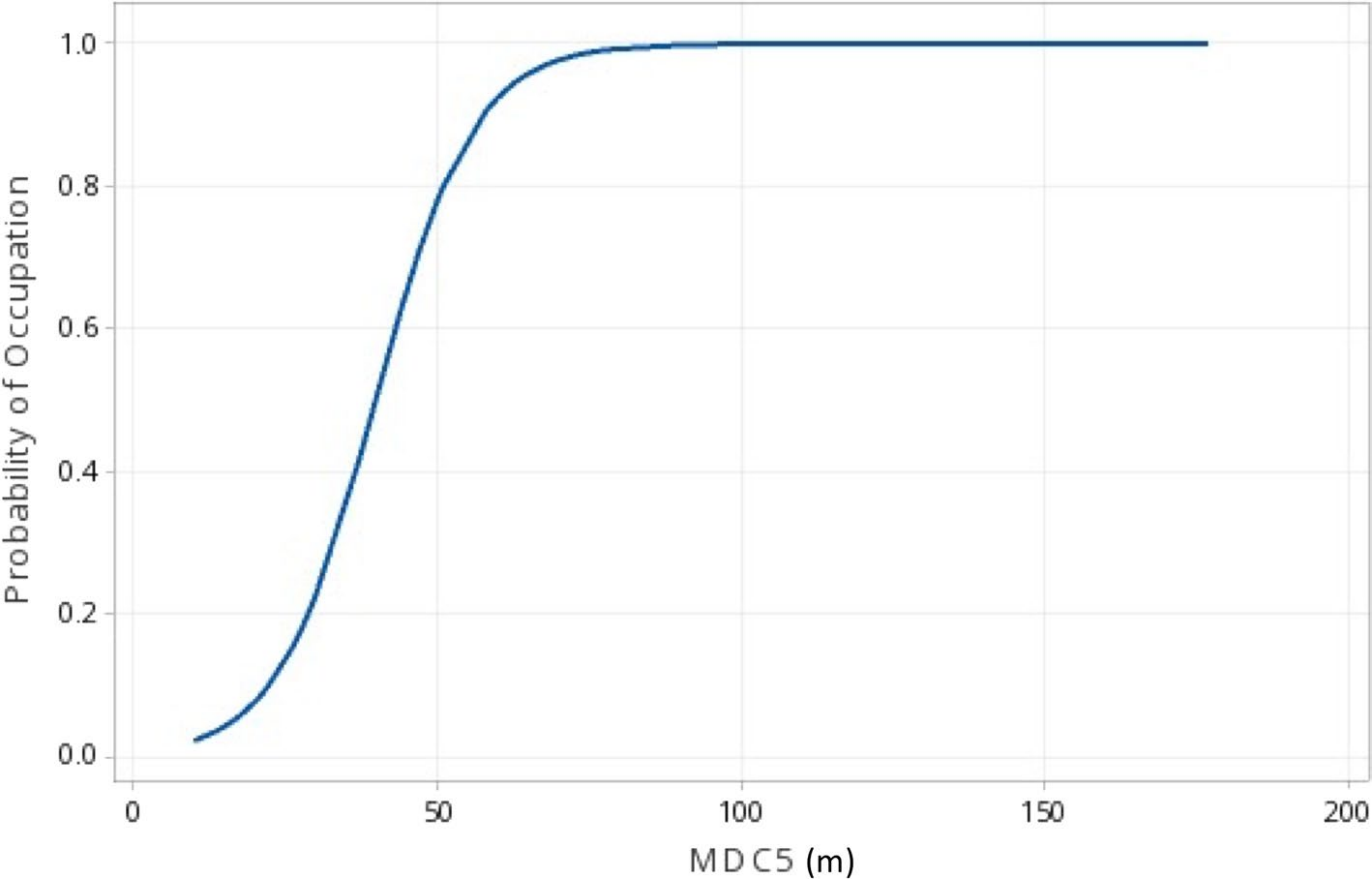
Guild 5 (DUGI1) predicted probability of threshold occupation vs. functional patch size (MDC [m]) of Patch Type 5 (deciduous understory). Probability of occupation as a function of patch size is shown between 2 occupancy levels—unoccupied (0) and occupied but not including the largest species (1). Occupied patches were significantly larger (x̅_MDC_ = 73 m) than unoccupied patches (x̅_MDC_ = 25 m, t-test, p < 0.001, n = 72). See Table 4 and OR 18

Successional changes in avian species occurrence, richness and density on the 23 sites were analyzed previously and were strongly associated with the changing amount and vertical distribution of leaf area during succession (Keller et al. 2003, tables 5–7, figs 4–7). Year-to-year (1977–1981) changes in leaf area on individual clearcuts and oldfields were modeled using the Mitscherlich Curve. The Mitscherlich Curve and its inverse also were used to derive estimates (for non-air photo years) of changes in the sizes of MDC and DEAC, respectively, as well as TOTAMT. This was reasonable because the changes in MDC and DEAC values between the 1977 and 1980 air photos were essentially measures of the rate of canopy closure (DS Robson, Cornell Biometrics Unit, pers. comm.). This modeling did not apply to the 2 mowed oldfields and 2 of the 4 forests (OR 3), for which landscape component coverage was unchanged between the 1977 and 1980 air photos. Between photo years, new patches of Types 1, 11, and 13 formed on the other 2 forest sites as a result of canopy openings due to treefalls.

We note that although site surveys in consecutive years could arguably reduce sample independence due to factors such as avian site fidelity, the great majority (19 of 23) of sites were undergoing quantifiable changes in study covariates (patch composition, functional size, and amount) that were biologically relatable to and testable against concomitant changes in avian species occurrence and richness. Moreover, studies of autocorrelation have concluded that if the resolution and scale of analysis and the resolution and scale of species response are well matched, and the analysis includes relevant covariates acting at that resolution and scale, then the variance due to autocorrelation, as a proportion of total explained variance, is likely to be much smaller than it appears (Diniz-Filho et al. 2003, 2007; Knegt et al. 2010). As Thogmartin et al. (2004, p. 1770) noted, “Ideally, spatial structuring in the model would be unnecessary, given the inclusion of a proper set of environmental covariates defining the spatial relatedness between counts.” Our image resolution (< 0.75 m), scale of analysis (intraterritory), and guild-specific covariates are consistent with these interpretations.

MDC/DEAC and TOTAMT were highly correlated (r = 0.6 to 0.988). Therefore, we tested the predictive power of each variable separately in Stepwise Multiple Regression analyses (JMP Pro 15.0.0 SAS Institute, Cary, NC). Similarly, circle area (MDC, DEAC) and diameter (MDCA, DEACA) were highly correlated (r > 0.9) and each was entered separately in regression trials. This resulted in a total of 57 trials of species richness (3 for 13 multispecies guilds) or occurrence (3 for 6 single species guilds).

Variation in survey site size raised the question of statistical validity of comparing MDC/DEAC and TOTAMT across sites. Although size of local landscapes may be fixed across samples in tests of the HAH, patch type total amount can vary between landscapes depending on the question being asked. Here, although amount of patch type T varied across sites, it was fixed on each site for a given survey year (Fahrig 2013, fig. 7b). This allowed direct within-year comparison of MDC/DEAC with TOTAMT as predictors of threshold guild occupancy and richness. SITESIZE and NUMHAB also were included to test whether the size of local landscapes or observed heterogeneity of each patch type T within a local landscape influenced species richness of guilds beyond the effects of functional patch size or total amount. We entered SNAGTOT in regressions for 4 guilds (2, 11, 17, 18) that included cavity nesters. Each model trial tested all possible combinations of predictors entered for that guild and included all survey years (N) for any site on which any amount of patch type T occurred for the associated guild during the study.

We calculated a variance inflation factor (VIF) for each set of proposed predictors to identify multicollinearity among predictors. We detected no substantial multicollinearity (VIF ≥ 5.0) among predictor variables for any of the selected models. We corroborated model selection using AIC and Mallows Cp statistic, applying the criterion that all retained variables had to explain a significant (*P* < 0.05) portion of model variance. The variable that explained the highest proportion of variance in a multivariate model is referred to as the primary variable. Effect size of each retained variable in the model was assessed using the F Ratio statistic in JMP Pro 15.0.0 (SAS Institute, Cary, NC).

We modeled threshold patch occupancy using binary logistic regression in Minitab. Patches of type T were categorized as present but unoccupied (0) or occupied (1). Occupied patches were those supporting single species guilds or for multispecies guilds, any species combination without the largest species present. We performed ordinal logistic regression in Minitab to test for the nonrandom appearance of the largest species in each of the 13 multispecies guilds. Patches of type T were categorized as existing but unoccupied (0), occupied by the guild but without the largest species present (1), and occupied by the largest species in the guild (2). Direct comparisons of patch size differences between each of the 3 categories for each guild were made using a t test. Because these latter 2 logistic regression analyses examined threshold occupancy, not species richness (i.e., were based on patch size or amount), we did not include within patch heterogeneity (NUMHAB) among predictor variables. All other predictors remained the same. Lastly, we tested for the nonrandom occurrence of the initial guild member based on body size using a Rank Sum test (OR 12, DS Robson, Cornell Biometrics Unit, pers. comm.).

## Results

### Guild species richness

Functional patch size was the sole/plausible sole (n = 8 models) or primary (n = 8 models) predictor variable in multivariate regression models of species richness for 15 (79%) of the 19 guilds (Table 3, Fig. 6, OR 13, OR 14a, OR 15a), including 11 of 13 multispecies guilds. Total patch amount was a plausible sole predictor in a model for the ruby-throated hummingbird (Guild 19), was primary in 1 other model and was included in models for 6 (32%) of 19 guilds (OR 14b, OR 15b, OR 16b). Within patch heterogeneity (NUMHAB: Guilds 9, 13, 14), snag density (SNAGTOT: Guild 18), and the size of survey sites (SITESIZE: Guild 12) were sole (OR 16c) or primary predictors of species richness for 5 guilds. They were included in 14 of 23 plausible models, most frequently as secondary or tertiary predictors. Where primary, functional patch size explained a high proportion of model variance (x̅ = 86%, n = 8). All other primary predictors explained a substantially lower portion of model variance (x̅ = 54%, n = 5, OR 17).

**Table 3:** Most parsimonious (AIC) multiple linear regression models of avian guild species richness on 23 clearcuts, oldfields, and forests at the Connecticut Hill WMA in central New York

Guild	Multiple linear regression models	R-SQ
1) DLGI1 (8^a^)	0.552 + 0.027*MDC1 + 0.327*NUMHAB1^b^	0.647*** ^c,d^
2) DMGI (6)	− 1.503 + 0.0169*MDC2 + 0.711*NUMHAB2	0.391***
	− 0.350 + 0.0032*TOTAMT2 + 0.684*NUMHAB2− 0.119*SITESIZE^e^	0.410***
3) DHGI1 (2)	0.0068 + 0.0112*MDC3	0.835***
	− 0.082 + 0.011*MDC3 + 0.014*SITESIZE^e^	0.841***
4) DTGI1 (7)	− 0.059 + 0.128*MDC4 + 0.047*SITESIZE	0.740***
5) DUGI1 (5)	− 0.643 + 0.0196*MDC5 + 0.1459*NUMHAB5	0.721***
6) CHGI1 (4)	0.017 + 9.01E− 05*MDCA6	0.836***
7) OTGI1 (5)	− 0.047 + 0.0003*MDCA7	0.615***
8) CTGI2 (2)	− 0.218 + 0.026*DEAC8	0.307***
9) DLGI2 (9)	0.0143 + 0.036*DEAC9	0.603***
	0.262 + 0.279*NUMHAB9 + 0.0015*TOTAMT9^e^	0.615***
10) DLSI2 (1)	− 0.065 + 0.0001*DEACA10 + 0.066* NUMHAB10	0.352***
11) MHSI2 (2)	− 0.0887 + 0.0001*DEACA11 + 0.0151*SNAGTOT	0.511***
12) DBGI2 (1)	0.0687− 0.024*SITESIZE + 0.0017*TOTAMT12 + 0.069*NUMHAB12	0.380***
13) MLGI2 (1)	− 0.011 + 0.277*NUMHAB13	0.721***
14) DTGI2 (2)	0.7308 + 0.0821*NUMHAB14 + 0.088*SITESIZE + 0.001*TOTAMT14	0.718***
15) MMGI2 (1)	0.039 + 0.0002*DEAC15	0.743***
16) CHGI2 (1)	0.071 + 0.0002*DEACA16 + 0.02*SITESIZE	0.304***
17) DBGI1 (2)	− 0.089 + 0.007*MDCA3	0.712***
18) DBPI (5)	− 0.0138 + 0.0366*SNAGTOT + 0.001*TOTAMT3	0.727***
19) DLHN2 (1)	0.165 + 0.0008*TOTAMT10	0.098**
	0.136 + 7.21e− 5*DEACA10^e^	0.094**

Three model trials were completed for each guild. Each included only 1 of the following predictors of patch type T amount/size for the associated guild (Table 1, OR 6): (1) TOTAMT or (2) MDC/DEAC or (3) MDCA/DEACA. Each trial also included within patch type T heterogeneity (NUMHAB) and the size of each local landscape surveyed (SITESIZE, OR 3). Total snags ha-2 (SNAGTOT) was entered for guilds 2, 11, 17, and 18 which included cavity nesters. Each trial tested all possible predictor combinations

^a^Potential number of species in the guild; guild classification and composition in OR 5 and OR 6, respectively

^b^Variable definitions in Table 2. Predictor variable in **bold** for ease of variable identification and comparison

^c^**P < 0.01, ***P < 0.0001

^d^All N = 97 except guilds 8 and 11 (N = 84) and guild 15 (N = 45)

^e^∆AIC < 2.0 both models plausible

### Threshold patch occupancy

Functional patch size was the sole/plausible sole (n = 11) or primary (n = 2) predictor in binary logistic regression models of threshold occupancy for 13 (72%) of the 18 guilds for which regressions were calculable (Table 4, Fig. 7), including 10 of 12 multispecies guilds. Total patch amount was the sole / plausible sole predictor for occupancy of 5 guilds (28%; OR 16d) and was primary in 1 model (5.5%). Site size was a relatively poor (AUC < 0.7) sole predictor for the ruby-throated hummingbird (Guild 19) and was included in 3 other models. Snag density was the sole variable in threshold occupancy models for bark probers (Guild 18) and bark gleaners (Guild 17). Threshold occurrence of grassland birds (Guild 7) was not calculable with binary logistic regression but was well predicted by functional patch size (MDCA7) using categorical linear regression (R^2^ = 0.748, *P* < 0.0001, N = 62, OR 18).

**Table 4:** Most parsimonious (AIC) multivariate binary logistic regression models of threshold guild occupancy on 23 clearcuts, oldfields, and forests at the Connecticut Hill WMA in central New York. Categories are size of present but unoccupied (PBU) type T patches (0) and size of occupied patches (1), either for single species or for multispecies guilds without the largest species present (O w/o LG)

Guild	Binary logistic model	Predictor *p*^a^	R SQ	AUC^b^	N
1) DLGI1	− 0.342 + 0.067*MDC1	NS (0.148)	0.189^c^	0.739	33
2) DMGI	− 2.506 + 0.045*MDC2 + 0.147*SITESIZE	*** *	0.307	0.826	83
3) DHGI1	− 2.31 + 0.041*MDC3	*	0.092	0.786	61
	− 2.217 + 0.023*TOTAMT3^d^	*	0.106	0.637	61
4) DTGI1	− 0.59 + 0.176*MDC4	NS (0.073)	0.130	0.769	62
5) DUGI1	− 4.94 + 0.124*MDC5	***	0.584	0.955	72
6) CHGI1	− 4.25 + 0.0224*TOTAMT6	*	0.644	0.979	62
	− 4.92 + 0.004*MDCA6^d^	*	0.613	0.949	62
7) OTGI1	**NC**^e^		^e^		62
8) CTGI2	− 6.34 + 0.168*DEAC8	***	0.376	0.857	62
9) DLGI2	− 0.232 + 0.043*DEAC9	*	0.102	0.714	68
	− 0.667 + 0.043*TOTAMT9^f^	NS (0.056)	0.374	0.878	68
10) DLSI2	− 3.83 + 0.0603*DEAC10	***	0.203	0.760	79
11) MHSI2	− 5.38 + 0.0009*DEACA11	**	0.464	0.935	54
12) DBGI2	− 2.42 + 0.0431*TOTAMT12− 0.629*SITESIZE	** **	0.470	0.927	58
13) MLGI2	− 3.19 + 0.0242*TOTAMT13	***	0.487	0.873	37
14) DTGI2	− 5.01 + 0.2244*DEAC14− 0.892*SITESIZE	** *	0.655	0.972	39
15) MMGI2	− 4.38 + 0.0986*DEAC15	**	0.511	0.914	27
16) CHGI2	− 5.53 + 0.00249*DEACA16	**	0.312	0.904	52
17) DBGI1	− 6.62 + 0.1694*SNAGTOT	*	0.469	0.965	67
18) DBPI	− 4.36 + 0.3116*SNAGTOT	***	0.738	0.947	73
19) DLHN2	− 1.72 + 0.1204*SITESIZE	*	0.070	0.657	79
	− 1.32 + 0.0032*TOTAMT10^d^	*	0.058	0.635	79

Candidate model variables entered are as in Table 3, except for NUMHAB. See OR 19 for PBU vs. O w/o LG patch size comparisons. See OR 5 and OR 6 for guild abbreviations and members

^a^*p < 0.05, **p < 0.01, ***p < 0.001, NS not significant, NC not calculable

^b^Area under the receiver operating curve

^c^All model R-SQ *P* < 0.05 except as noted (Guilds 1, 4, 9 [TOTAMT])

^d^∆AIC < 2.0 both models plausible

^e^Binary linear regression model for MDC7: R^2^ = 0.748, *P* < 0.0001, N = 62; OR 18

^f^Both models plausible based on separate criteria: significant *p* value of DEAC9 vs substantially lower AIC of TOTAMT9

Corroboratively, in 17 of 18 testable direct comparisons, the functional size of occupied type T patches was significantly larger (average MDC or DEAC) than unoccupied patches (comparison ab for multispecies guilds, OR 19, all *P* < 0.03), the exception being patch size for the ruby-throated hummingbird (Guild 19). Predicted threshold patch size and the increase in patch size to effect occupation by additional species tended to be larger for mature forest-associated guilds (OR 20). Across all guilds, MDC/DEAC differed significantly (t-test, *P* < 0.0001, N = 1038) between unoccupied patches (x̅ = 25 m), patches occupied by all but the largest guild member (x̅ = 62 m), and patches that supported the largest species (x̅ = 100 m, OR 19). Additionally, the smallest species by weight was the species most likely to occur when only one guild member was present in 8 of the 13 multispecies guilds (OR 21). Seven of these cases were significant.

Distributions of all patch types (T) included sites that contained one or more small patches of type T but did not support any members of the associated guild. For example, although most of the early successional sites (CCA, CCB, OFF, OFI, and OFA, Table OR 3) contained some open grass patches (Patch Type 7), no grassland birds (Guild 7) occurred on sites with MDC’s of open grass less than 73 m (OR 18).

### Larger species in larger patches

**Fig. 8:**
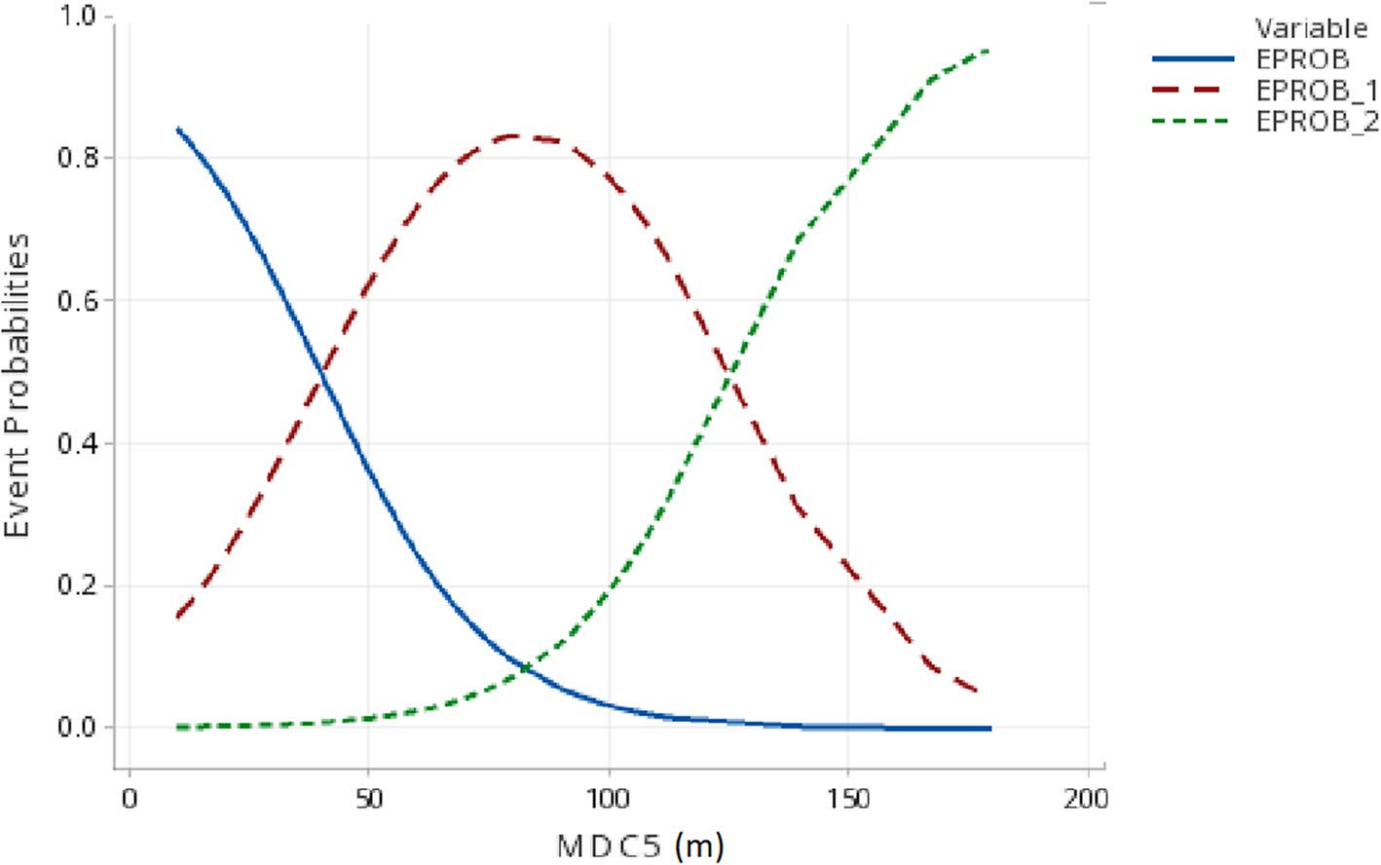
Guild 5 (DUGI1) predicted event probabilities vs. patch size (MDC [m]) of deciduous understory (Patch Type 5). Patch categories are unoccupied (EPROB), occupied by all but the largest species (EPROB_1), and occupied by the largest species (EPROB_2). Patches occupied by the largest species were significantly larger (x̅_MDC_ = 159 m) than patches occupied by smaller guild members (x̅_MDC_ = 73 m, t-test, p < 0.001, n = 66). See Table 5, OR 19

Functional patch size was the sole (n = 7) or primary (n = 1) predictor of patch occupancy by the largest species in ordinal logistic regression models for 8 (62%) of the 13 multispecies guilds (Table 5, Fig. 8—event probability 2). Total patch amount was the sole (n = 2) or primary (n = 2) variable in 4 models (31%). However, functional patch size (MDC/DEAC) also was a highly significant predictor for 3 of the 4 guilds where TOTAMT represented the most parsimonious model (Guilds 2, 9, 14: all *P* < 0.001; model not calculable for Guild 17). Snag density was the sole predictor for bark probers (Guild 18) and was included in one other model. Site size was a secondary variable in 2 models. In 7 of 11 direct comparisons between the largest guild member and smaller guild members, the largest species occupied significantly larger patches than smaller species (OR 19, comparison bc, *P* < 0.03, t test). However, comparisons for Guilds 8 and 11 revealed that the larger of the 2 species in each guild actually occupied smaller patches, on average, than the smaller guild member.

**Table 5:** The most parsimonious (AIC) multivariate ordinal logistic regression models of occurrence for the largest guild member of the 13 multispecies guilds. Occupancy categories are size of present but unoccupied type T patches (0), the size of occupied patches without the largest species present (1), and size of patches occupied by the largest guild member (2)

Guild	Intercepts k1, k2^a^	Ordinal Logistic Model^b^	Predictor *p*^c^	R-SQ	N
1) DLGI1^d^	0.148, 3.077	**−**0.0442*MDC1−0.2377*SITESIZE	*** **	0.415^e^	88
2) DMGI	**−**0.001, 4.152	**−**0.0059*TOTAMT2 + 0.0606*SNAGTOT	*** *	0.407	97
3) DHGI1	2.585, 5.153	**−**0.060*MDC3	***	0.775	81
4) DTGI1	0.466, 5.717	**−**0.171*MDC4	***	0.415	79
5) DUGI1	2.238, 7.051	**−**0.0559*MDC5	***	0.756	97
6) CHGI1	4.917, 102.99	**−**0.0041*MDCA6	*	0.779	70
7) OTGI1	7.311, 13.48	**−**0.1121*MDC7	**	0.773	63
8) CTGI2	2.591, 4.643	**−**0.0827*DEAC8	***	0.245	82
9) DLGI2	**−**1.333, 3.247	**−**0.003*TOTAMT9	***	0.278	79
11) MHSI2	5.258, 6.808	**−**0.63*DEAC11	***	0.395	61
14) DTGI2	**−**1.682, 0.812	**−**0.0134*TOTAMT14 + 0.6253*SITESIZE	*** ***	0.629	79
17) DBGI1	5.865, 6.614	**−**0.0084*TOTAMT3	***	0.810	81
18) DBPI	3.547, 8.32	**−**0.2317*SNAGTOT	***	0.785	81

Candidate model variables are the same as those in Table 4. See OR 19 patch size comparisons among guild occupancy levels

^a^Intercepts for the first (k1) and second (k2) events of 3 response categories (occupancy levels = 0, 1 and 2)

^b^Categorical linear regressions of patch size (MDC/DEAC) alone were highly significant (*P* < 0.001) for all guilds

^c^*p < 0.05, **p < 0.01, ***p < 0.001

^d^See OR 5 and OR 6 for guild abbreviations and species. See Table 1 for patch type T–guild associations

^e^All models *P* < 0.001

Species addition by increasing body size was significant (*P* < 0.001) across all species for the deciduous mid-canopy gleaners (Guild 2, OR 21), an assemblage with distinct body size separation among species. Populations of cuckoos, the largest mid-canopy gleaners, occurred on 9 of 11 young clearcuts (CCA and CCB, n = 16 site years) where the MDC of deciduous mid-canopy (Patch Type 2) had developed to at least 80 m (x̅ = 116 m) by 1980–1981. They occurred on no clearcuts with mid-canopy patches smaller than this (x̅ = 67 m, *P* < 0.01, n = 16, Rank Sum Test).

Additional examples of body size differences in patch occupation included the 3 largest members of Guild 9, the brown thrasher, northern cardinal, and yellow-breasted chat, which occurred in shrub-opening edge (Patch Type 9, Fig. 4A). These species, which range from 1.8 to over 6 times as large as any other guild members, had 13 occurrences in a total of 11 of 97 survey years (OR 19). Sites that supported these larger species had a DEAC for shrub-opening edge > 50 m and in 9 of 13 occurrences, the DEAC was > 80 m (x̅ = 84 m). In contrast, the average diameter of this patch was significantly smaller on sites where the guild occurred without these largest species (x̅ = 57 m, *P* < 0.006, t test). These 3 species always occurred in addition to smaller guild members that were more widely distributed. Similarly, the scarlet tanager, in addition to never being the first member of its guild to appear on a site (OR 21, DHGI1), never held a territory on sites with a stand-scale high-canopy MDC of less than 480 m.

Overall, in 12 of the 13 multispecies guilds, the smallest species in the guild was the species most likely to occur when only one guild member was present (Guilds 1, 2, 3, 6, 7, 8, 11, 14) and/or larger guild members occurred only in larger patches (Guilds 1, 2, 3, 4, 5, 6, 9, 14, 18).

### Relationships of site size and within patch heterogeneity to species richness

In addition to its infrequent inclusion in regression models of species richness (Table 3), SITESIZE, a measure of broader cover types (OR 3) that typically include multiple patch types (T), was significantly correlated with richness of only 8 guilds (OR 22). All of these correlations with site area were weaker (x̅_r_ richness with SITESIZE = 0.218) than those for the same guild with MDC/DEAC. Instead, SITESIZE was more strongly associated with broader scale assemblage-level metrics such as total species richness (R^2^ = 0.362), guild richness (R^2^ = 0.157), and at an intermediate assemblage level, the species richness of shrubland birds (R^2^ = 0.403) (OR 23, all *P* < 0.0001). Regressions of species richness with NUMHAB, the measure of within patch heterogeneity, were significant for 8 of the 13 multispecies guilds (1, 3, 4, 7, 9, 14, 17, 18, [x̅_R2_ = 0.327], OR 24).

## Discussion

The HAH predicts that species density, the richness of a species group associated with a sample in patch type T, should be more highly correlated with the total amount of type T within a local landscape than with the size of the individual patch of type T surrounding that sample (Fahrig 2013; Watling et al. 2020). However, considering conservation or management of territorial species, the HAH does not account for influences on species distributions due to patch attributes such as shape, internal uniformity, or minimum critical (threshold) size (Keller 1986), which are often limiting in human-dominated landscapes (Gibson et al. 2013; Mathews et al. 2014; Keller 2021). When these additional patch attributes were considered, counter to HAH prediction, species richness for 15 (79%) of the 19 avian guilds was better explained by functional patch size (MDC/DEAC, Table 3, OR-17) based on optimal territory shape than by total patch amount (2 guilds, 11%). Three other biologically interpretable predictions of threshold patch occupation consistent with functional patch size also were well supported by the data.

First, no guild member associated with patch type T should be present in a local landscape if no single patch of type T is functionally large enough to support a territory (Fig. 1), regardless of the total amount of type T in the landscape (Table 4, OR 19, Figs. 5, 6, OR 18). Unlike many forest interior species reported to be area sensitive (Robbins et al. 1989; Whitcomb et al. 1981; Smith et al. 2011), species associated with ephemeral biotopes such as early successional forests, successional oldfields, and forest canopy gaps (e.g., guilds 1, 4, 8, 9, 10, 13, 14, 15, 19) appear especially adapted to locating small isolated patches of habitat within larger matrices of other cover types (Schlossberg and King 2009; Perkins and Wood 2014). Yet, despite the association of many shrubland birds with small ephemeral patches, threshold patch sizes have been identified below which they will not occupy a site (Costello et al. 2000; Moorman and Guynn 2001; Rodewald and Vitz 2005; Roberts and King 2017). Note, the effect sizes (partial regression coefficients) for MDC and DEAC in species richness models are small (Table 3). We attribute this to the small territory sizes, typically 0.4 to 2 ha, of the mostly passerine species studied. A 0.4 ha territory only has a MDC of approximately 70 m and even small increases in circle diameter produce relatively large changes in circle area (i.e., potential territory size) associated with additional species (OR 19, OR 20; Fig. 6, OR 13, OR 14a).

Although the stronger association of guild species richness with functional patch size than with total patch amount is a reasonable expectation evolutionarily for mobile territorial species associated with ephemeral and/or isolated habitats, richness of most guilds associated with older forest stages also was associated more strongly with functional patch size (Table 3). Similarly, the other 2 patch size predictions—smallest species occur first (OR 21) and larger species occur only in larger patches (Table 5, Fig. 8)—were supported across multispecies guilds regardless of the successional stage of their associated patch type. Support for these latter 3 functional patch size-based predictions is thus both inconsistent with the HAH and consistent with an energetics driven body size-patch size relationship where patch shape (Covich 1976) strongly influences the minimum patch size at which increasingly larger animals are able to establish all-purpose territories (Schoener 1983; Keller 1986; Lindenmayer et al. 1999; Beier et al. 2002). Other studies have noted both large patch and small patch associations/dependence for multiple taxa (MacDonald et al. 2021; Deane 2022).

Because the HAH attributes observed species richness to increased sampling area, data used to test the hypothesis (i.e., species richness in a sample plot and total habitat amount) do not contribute to an understanding of nonrandom presence or absence of assemblage members (Haefner 1981; Adams 2007; Püttker et al. 2015; Fletcher et al. 2018; MacDonald et al. 2021). Thus, although TOTAMT was the primary variable in a plausible 3-variable model of richness for Guild 2 (Table 3), the simpler 2-variable model with MDC is more consistent with the observed addition of progressively larger species to this guild as intraguild richness increases (OR 21). Ecologically, the body size-functional patch size explanation of threshold occupation provides insight beyond assemblage richness to the pattern of initial appearance, species addition, and ultimate species composition of guilds (Fig. 1b), and the biotope-level assemblages of which they are a part (Banks-Leite et al. 2012; Maglioli et al. 2015; Rösch et al. 2015; Haddad et al. 2017; Keller 2021). This also suggests the potential basis for top-down body size-based species losses due to fragmentation associated with habitat loss, especially where newly converted matrix is inhospitable to immigration or emigration (e.g., Gibson et al 2013). Some prediction inconsistencies with threshold patch size remain, however. The appearance of snag density (SNAGTOT) as sole predictor of threshold occupancy for Guilds 17 and 18 (Table 4) illustrates how limitations other than patch size or total amount can dictate species composition and richness. The potential influence of weather or nest site availability, stronger correlations of species richness with total patch amount for 5 guilds (OR 22), and the need for further refinement of guild-patch associations are discussed in OR 25.

### Matching species groups to patch types— the problem of within-patch heterogeneity

Interpretations of the influence of patch size, total amount, and spatial arrangement on species richness rely heavily on how precisely species groups are defined, and on the composition and uniformity of associated patch types (Fahrig 2013, p. 1656; MacDonald et al. 2021, p. 13). For example, combining the more than 25 shrubland species, although producing a strong relationship (OR 23) of shrubland bird species richness with the size of more general cover types (SITESIZE), obfuscates the within-site habitat heterogeneity with which richness is strongly associated when the landscape is examined at HR (OR 24, Table 3). Potentially more importantly, it masks assemblage composition (i.e., which guild and species subsets of shrubland birds occur on each local landscape) and the importance to management decision-making of understanding finer-grained species-habitat relationships (Banks-Leite et al. 2012; Hanski 2015; Collins et al. 2017; Fletcher et al. 2018). Examining solid and edge habitat associations of shrubland birds at a within-territory scale using HR stereoscopic imagery suggested species assignment to 10 different species groups (Guilds 1, 2, 4, 5, 8, 9, 10, 14, 15, 19; OR 6) associated with 9 restrictively defined patch types (OR 8) that collectively represented the range of vegetation composition and structural heterogeneity across early successional upland forests and oldfields in this Northeastern USA landscape.

As with richness of the shrubland bird assemblage, richness of all species combined and *guild* richness on each site were significantly associated with SITESIZE (OR 23). This is consistent with the concept that as patches of more general cover types increase in size, they are more likely to contain threshold- or larger-sized guild-specific patches (Table 4, Fig. 6, OR 13, OR 14a, OR 15a—occupied vs. unoccupied patch sizes). Yet, the weak correlations of intraguild richness with SITESIZE (OR 22) indicate that occupiable guild-specific patches are not distributed proportionally to the size of more broadly defined cover types in which they occur (Robbins 1979, p. 199; Wiens 1989, p. 388 and fig. 1; May et al. 2019). As a result, loss of both patch type and species distribution information within broad cover types viewed at LR and assemblages characterized by richness alone may misinform conservation or management decision-making (OR 26).

These relationships highlight the individual distribution of species (Gleason 1926) and suggest that correlations of species richness with total habitat amount are consistent with the HAH because summing the area of all patches captures heterogeneity inherent in larger spatial extents (OR 24; Rahbek and Graves 2001; Kallimans et al. 2008; Bar-Massada et al. 2012; Rösch et al. 2015). Even at the relatively small scale of patches studied here, this is evidenced by the inclusion of the measure of within patch heterogeneity (NUMHAB) in models of species richness for 8 guilds (Table 3). Geographic heterogeneity also is a plausible explanation for the consistent observation that a set of small patches supports more species than a set of fewer large patches (Seibold et al. 2017; Fahrig 2020, 3.3.4). By enabling identification of more specific patch types, HR GIS imagery also facilitates exclusion of landscape elements or cover types that are unused (i.e., non-type T = nonhabitat) by the focal species or group (Goetz et al. 2010; Tattoni et al. 2012; Vogeler et al. 2013; Keller and Smith 2014; Gaston et al. 2017). The precision with which species groups are defined, and patches defined and delineated also may affect interpretations of the influence of other patch attributes potentially important to conservation such as interpatch distance, connectivity, and matrix quality (Smith et al. 2002, 2003; Arponen et al. 2012; Haddad et al. 2017; Thompson et al. 2017). Collectively, these results suggest that analyses of habitat loss or fragmentation conducted at landscape scales should incorporate at least a subset of HR image analysis across study areas using more restrictively defined patch types to better assess the population distribution of taxa of conservation or management concern (Bombi et al. 2019; MacDonald et al. 2021).

### Energetic effects of patch shape and perforation on functional patch size and threshold occupancy

The HAH postulates that patch size effects on species richness are simply habitat amount effects because larger patches contain more habitat (Fahrig 2017), which as noted above, also may be the result of unmeasured within patch heterogeneity. Saura (2020, p. 13, figs. 1–4) attributed this effect to patch shape (termed “configuration”), noting that, “When all habitat is found in a single and compact habitat patch, there will be more (HAH sample) sites with a high habitat amount in their local landscape,” resulting in a higher species response value (i.e., occurrence or richness). The relationship of territory size to body size coupled with the circle as optimal territory shape suggests the physical basis for this correlation.

Similarly, previous studies of grassland birds reported stronger relationships of occurrence and richness with patches exhibiting lower perimeter area ratios (i.e., where patch shape approached a circle) than with patch size (Helzer and Jelinski 1999; Davis 2004). Here, grassland birds only occurred on sites with open grass MDC’s larger than 73 m, regardless of total area of open grass on the site. Additionally, grass patches often were perforated with non-grass landscape components (Fig. 5, largest grass patch). Thus, for a territorial species associated with a solid patch type T, although the total amount of a single patch of type T may exceed the minimum territory size of the species, either of two conditions may reduce patch functional size below that for occupation by that species or, at a larger spatial scale, reduce its potential species richness.

First, if a solid patch of type T is overly perforated with non-type T landscape components, its internal spatial arrangement may be too energetically inefficient to support occupation or maximum species richness (Fig. 3a, Table 4, OR 19, OR 20; Beier et al. 2002). Second, even if homogeneous, the shape of a patch with a high perimeter to area ratio (e.g., linear, amoebic) reduces its functional size to something less than that of a circle for the same area (Fig. 2; Keller 1986; Helzer and Jelinski 1999; Saura 2020, fig. 2A vs. 2E and 2H). This is true until the patch is multiple times the diameter of the largest territory of interest (i.e., until the patch is able to support a metapopulation). At that point, territory shape and locations relative to one another are unconstrained by patch shape, suggesting that at landscape scales patch shape may be unimportant (Simberloff 1986; Andren 1994; Fahrig 1997; Flather and Bevers 2002).

Yet, at a scale of effect of 2 km radius, negative fragmentation effects on Brazilian Atlantic Forest species were attributed to ecotone-scale edge density at intermediate (30–60%) and high (> 60) levels of remaining forest cover for animals and plants, respectively (Püttker 2020). This suggests that even when total amount of a focal patch type represents an area much larger than examined here, if patches composing the total are inefficiently shaped and/or perforated, they may not support a sufficient number of territories to sustain metapopulations of patch-interior species with larger territories or limited dispersal capability (Pfeifer et al. 2017). The authors recommended that even in highly forested landscapes, management should focus on reducing such edge effects. This argues that optimizing the shape of patches to increase functional patch size (MDC) by infilling (perforations in Fig. 3a vs. b) or addition (fig. 15.12, Keller 2021) via acquisition and/or restoration has importance at scales much larger than threshold occupation of passerines (Nol et al. 2005; Banks-Leite et al. 2011). For example, increasing functional patch size through shape optimization may have value at critical local extinction thresholds often associated with the transition from intermediate to low levels (< 30%) of habitat amount (Tambosi et al. 2014) where patch sizes are generally smaller (Ribeiro et al. 2009) and maintenance of metapopulations, particularly of larger species, is compromised (Poiani et al. 2001; Haskell et al. 2002; Daily et al. 2003; Maglioli et al. 2015; Bogoni et al. 2017).

Watling et al. (2020, p. 6) countered that “Large patches are important for species density because they contribute to high habitat amount, but they are no more important than a collection of multiple patches summing (to) the same total area in local landscapes around sample plots.” As demonstrated here (Table 5, OR 19), this will not be true if the largest available patch is too small for occupation by larger species in the focal group (Fig. 1a), either due to lack of overall size or energetically inefficient shape. In an acknowledgement of this possibility, Watling et al. (2020, p. 7) later noted that “some study regions may have been subject to anthropogenic disturbance for so long that assemblages are depauperate in species with large patch size requirements.” Thus, we agree that all habitat patches, regardless of size, are valuable for conservation (Watling et al. 2020) but suggest this concept is incomplete. Knowledge of assemblage richness without considering composition and natural history of species or species subsets that constitute richness may result in correspondingly incomplete conservation at any organizational level (Banks-Leite et al. 2012; Mathews et al 2014; Püttker et al. 2015; Fletcher et al. 2018; Valente and Betts 2018). The potential utility of functional patch size as a complimentary metric to total patch amount should be evaluated at larger spatial scales than those studied here.

## Conclusions

Patches larger than threshold territory size are essential to provide habitat for taxonomic assemblages that include territorial species. Functional patch size can be used to quantitatively assess the effects of patch size, shape or perforation on threshold habitat availability of species-scaled, organism-centric patch types. This information can assist practitioners in the design and defensible prioritization of conservation, restoration or enhancement options for focal taxa or biodiversity in general. We recommend that management or conservation strategies integrate the “all habitat patches are important” precept of the HAH with the concept of functional patch size.

### Supplementary Information

Below is the link to the electronic supplementary material.

Supplementary file1 (PDF 7199 KB)

## Data Availability

Not applicable.

## Data availability

Data will be made available upon reasonable request.
