# Supplementary material for: The importance of patch shape at threshold occupancy: functional patch size within total habitat amount
Source: Oecologia. 2023 Oct 10;203(1-2):95–112. doi: 10.1007/s00442-023-05453-3 (PMC10615919; doi:10.1007/s00442-023-05453-3)
Supplement: Supplementary file 1 — Supplementary file1 (PDF 7199 KB) [file 442_2023_5453_MOESM1_ESM.pdf]

THE IMPORTANCE OF PATCH SHAPE AT THRESHOLD OCCUPANCY: FUNCTIONAL PATCH SIZE WITHIN TOTAL HABITAT AMOUNT

Publication: - **Oecologia - Electronic Supplemental Materials**

Authors Jeffrey K. Keller and Patrick J. Sullivan - Department of Natural Resources and the Environment, Cornell University, Ithaca, New York 14853

J. Keller e-mail: habitat@epix.net

Current address: Habitat by Design, 74 Stagecoach Road, Pipersville, PA 18947

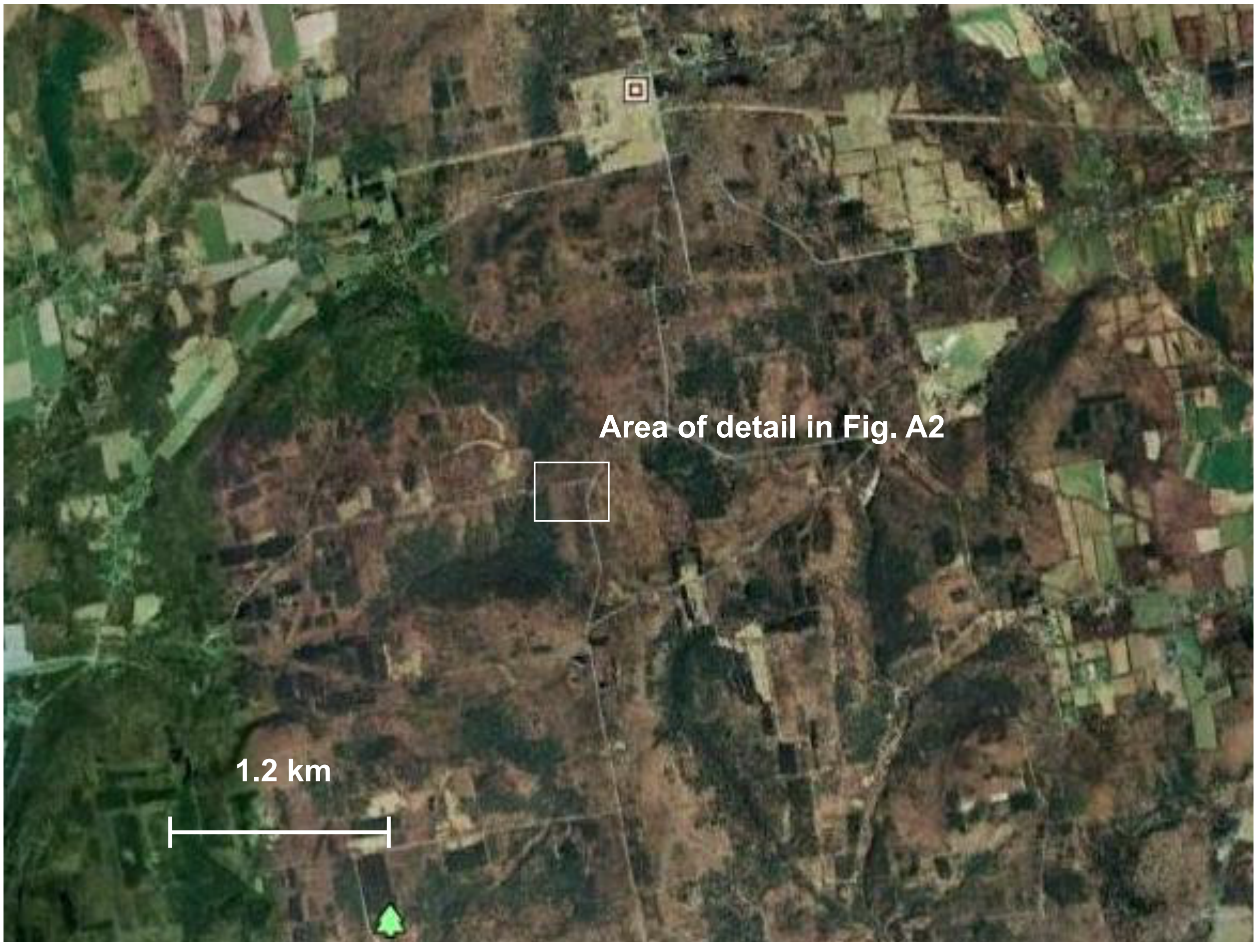

Online Resource (OR) 1 A 1:40000 (Landsat scale) view of the Connecticut Hill WMA in the Southern Finger Lakes region of central New York, USA illustrating landscape scale patches of different vegetation / land use types and the ecotone-scale edges between them. Notice how individual landscape components such as shrubs and smaller trees are not discernable at this image resolution. Source Google Earth May 1, 2007, New York GIS. From Keller and Smith 2014

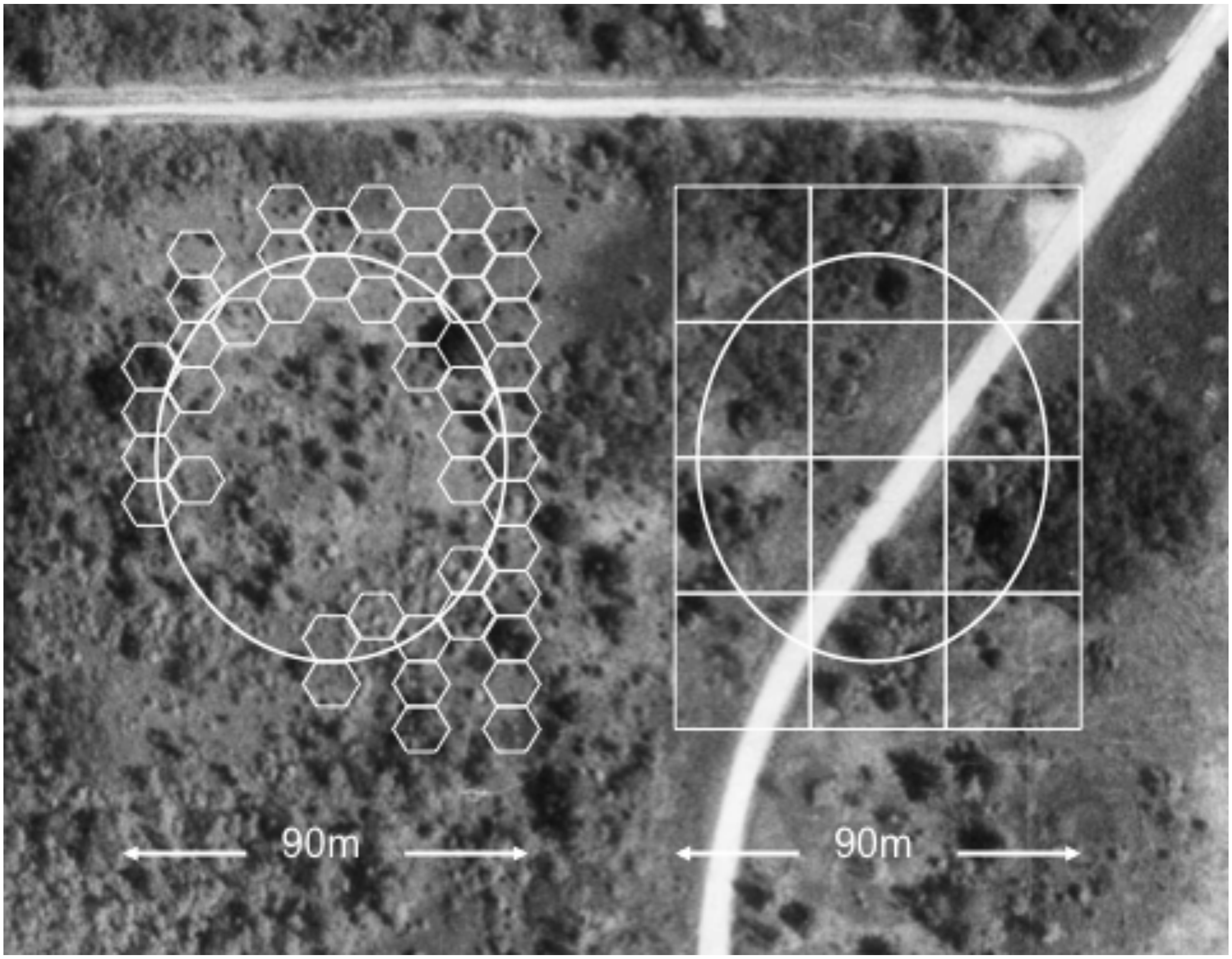

OR 2 (referenced as Fig. A2 in OR 1) Grids of two cell sizes, 100 m-2 hexagons and 900 m-2 squares, superimposed over nominal 0.81 ha passerine territories (ovals) on 1:2000 aerial photography with <0.75 m (NIIRS Level 6) resolution. Notice how this high-resolution image viewed at a large mapping scale allows identification of landscape components such as individual trees, shrubs, and small patches of open grass thought to be used in habitat selection by passerines. Note also the increased classification difficulties at the greater 30-m ground sampling distance (GSD = cell to cell center distance) of the Landsat-scale cells on high resolution imagery due to increased inclusion of multiple identifiable component types (e.g., deciduous sawtimber, deciduous saplings, deciduous shrubs, coniferous trees, bare ground, herbaceous cover, etc.) within a single 30x30-m (Landsat pixel equivalent) cell. The hexagonal celled grid was left partially devoid of cells to better illustrate the specificity of landscape component detail classifiable at this cell size (100 m-2). Source: 22 May 1977 aerial photography from an altitude of 1100 m using a Hasselblad camera and 70 mm black and white film. From Keller and Smith 2014

OR 3. Characteristics of the 23 sites studied at the Connecticut Hill Wildlife Management Area (WMA) between 1977 and 1981. From Keller et al. (2003).

Site	No. of years surveyed	Site age (yrs)	Stand Type[a]	Overstory trees left standing[b]	Size (ha)
CCA-1[c]	4	2-5	aspen-red maple	N	1.03
CCA-2	5	2-6	aspen	N	1.23
CCA-3	4	2-5	aspen-oldfield	N	2.80
CCA-4	4	2-5	jack pine/ash-red maple	N	3.15
CCA-5	4	2-5	aspen-red maple	Y	3.20
CCA-6	5	2-6	aspen-oldfield	Y	3.89
CCA-7	4	2-5	aspen-oldfield	Y	7.35
CCA-8	4	2-5	jack pine/ash-red maple	Y	10.46
CCA-9	4	2-5	jack pine/ash-red maple-oak	Y	13.82
CCA-10	4	2-5	aspen-oldfield	Y	24.15
CCB-1	5	4-8	NH-H/pincherry NH	Y	5.22
CCB-2	5	4-8	NH-H/pincherry NH	N	6.17
CCC-1	5	14-18	red maple-oak	N	2.20
CCC-2	2	24-25	red maple-white pine	Y	6.37
OFF-1	4	-	mowed field w/ perimeter shrub plantings	NA	5.36
OFF-2	4	-	mowed field w/ perimeter shrub and spruce plantings	NA	7.61
OFI	4	-	savannah w/ red maple and white pine	NA	3.92
OFA-1	4	48-51	dense shrub-sapling-pole-timber oldfield	NA	7.49
OFA-2	4	38-41	open shrub-sapling oldfield	NA	7.70
F-SGA	5	55-60	second growth deciduous pole-sawtimber	NA	10.00
F-NHHB	4	75	sawtimber northern hardwoods-hemlock	NA	8.89
F-NHHC	5	100	sawtimber northern hardwoods-hemlock	NA	8.47
F-NHHD	4	120	sawtimber northern hardwoods-hemlock	NA	9.76

a If precut and postcut stand types differed, the value to the left of the (/) is the precut stand type

b N = no; Y = yes; NA = not applicable

c Community Type: CC = clearcut; OF = oldfield; F = forest; SG = second growth deciduous; NHH = northern hardwood-hemlock. Increasing age within clearcut or forest: A-D
Oldfield Structural Type: F = frequently mowed (every 2-3 yr); I = infrequently mowed (every 5 yr); A = advanced woody invasion

OR 4. Weights of the 59 breeding bird species used in the data analysis. Bird weights were taken from a number of sources including Dunning (1984), Clench and Leberman (1978), Holmes and Sturgess (1975), and Stegeman (1955), and in some cases were averages of several sources.

Abbreviation	Common Name	Scientific Name	Weight (g)
YBCU	Yellow-billed Cuckoo	*Coccyzus americanus*	64.0
BBCU	Black-billed Cuckoo	*Coccyzus erythropthalmus*	51.1
RTHU	Ruby-throated Hummingbird	*Archilochus colubris*	3.0
AMWO	American Woodcock	*Scolopax minor*	197.0
YBSA	Yellow-bellied Sapsucker	*Sphyrapicus varius*	48.3
DOWO	Downy Woodpecker	*Dryobates pubescens*	26.7
HAWO	Hairy Woodpecker	*Dryobates villosus*	67.8
NOFL	Northern Flicker	*Colaptes auratus*	131.0
PIWO	Pileated Woodpecker	*Dryocopus pileatus*	287.0
GCFL	Great Crested Flycatcher	*Myiarchus crinitus*	31.0
EWPE	Eastern Wood Pewee	*Contopus virens*	12.8
ALFL	Alder Flycatcher	*Empidonax alnorum*	12.0
BHVI	Blue-headed Vireo	*Vireo solitarius*	16.8
REVI	Red-eyed Vireo	*Vireo olivaceus*	17.0
BCCH	Black-capped Chickadee	*Poecile atricapillus*	11.5
WBNU	White-breasted Nuthatch	*Sitta carolinensis*	20.5
BRCR	Brown Creeper	*Certhia americana*	8.2
HOWR	House Wren	*Troglodytes aedon*	11.2
EABL	Eastern Bluebird	*Sialia sialis*	32.1
VEER	Veery	*Catharus fuscescens*	32.1
HETH	Hermit Thrush	*Catharus guttatus*	29.9
WOTH	Wood Thrush	*Hylocichla mustelina*	48.9
AMRO	American Robin	*Turdus migratorius*	82.1
GRCA	Gray Catbird	*Dumetella carolinensis*	35.9
BRTH	Brown Thrasher	*Toxostoma rufum*	68.4
CEWA	Cedar Waxwing	*Bombycilla cedrorum*	36.0
PUFI	Purple Finch	*Haemorhous purpureus*	24.8
AMGO	American Goldfinch	*Spinus tristis*	13.1
GRSP	Grasshopper Sparrow	*Ammodramus savannarum*	17.5
CHSP	Chipping Sparrow	*Spizella passerina*	12.3
FISP	Field Sparrow	*Spizella pusilla*	12.5
DEJU	Dark-eyed Junco	*Junco hyemalis*	21.0
WTSP	White-throated Sparrow	*Zonotrichia albicollis*	26.5
SASP	Savannah Sparrow	*Passerculus sandwichensis*	18.0
SOSP	Song Sparrow	*Melospiza melodia*	20.5
EATO	Eastern Towhee	*Pipilo erythropthalmus*	42.5
YBCH	Yellow-breasted Chat	*Icteria virens*	23.4
BOBO	Bobolink	*Dolichonyx oryzivorus*	46.0
EAME	Eastern Meadowlark	*Sturnella magna*	145.0
RWBL	Red-winged Blackbird	*Agelaius phoeniceus*	61.0
COGR	Common Grackle	*Quiscalus quiscula*	111.0
OVEN	Ovenbird	*Seiurus auricapilla*	19.5
GWWA	Golden-winged Warbler	*Vermivora chrysoptera*	9.0
BWWA	Blue-winged Warbler	*Vermivora cyanoptera*	8.2
BAWW	Black-and-White Warbler	*Mniotilta varia*	10.4
NAWA	Nashville Warbler	*Leiothlypis ruficapilla*	9.2
MOWA	Mourning Warbler	*Geothlypis philadelphia*	11.2
COYE	Common Yellowthroat	*Geothlypis trichas*	9.5

MAWA	Magnolia Warbler	*Setophaga magnolia*	10.3
BLBW	Blackburnian Warbler	*Setophaga fusca*	10.4
CSWA	Chestnut-sided Warbler	*Setophaga pensylvanica*	11.1
BTBW	Black-throated Blue Warbler	*Setophaga caerulescens*	10.2
PRWA	Prairie Warbler	*Setophaga discolor*	8.3
BTGW	Black-throated Green Warbler	*Setophaga virens*	9.2
CAWA	Canada Warbler	*Cardellina canadensis*	11.3
SCTA	Scarlet Tanager	*Piranga olivacea*	27.0
NOCA	Northern Cardinal	*Cardinalis cardinalis*	42.5
RBGR	Rose-breasted Grosbeak	*Pheucticus ludovicianus*	43.9
INBU	Indigo Bunting	*Passerina cyanea*	13.5

OR 5. Guild classification system[a] for breeding birds at the Connecticut Hill WMA[b]. From Keller et al. (2003).

---

1) **General Habitat Affinity**
   D - deciduous
   C - coniferous
   M - mixed (deciduous and coniferous)
   O - open grassland

2) **Foraging Substrate**

For Foliage Gleaners	For Salliers	For All Others
L - low (0-3 m)	L - low (<6 m)	B - bark
M - medium (3-7 m)	H - high (>6 m)	T - terrestrial (0-2m) (in open canopy)
H - high (>7 m)		U - understory (in closed canopy)

3) **Foraging Technique**
   G - glean
   H - hover
   P - probe
   S - sally

4) **Prey Type**
   I - Insectivore (but see Secondary Prey Type)
   N - Nectarivore

5)

**Secondary Habitat Affinity**	**Secondary Prey Type (DTGI only)**
1 - solid patch	1 - insects
2 - edge patch	2 - insects and other invertebrates (particularly earthworms)

---

a An example of a guild defined using this classification system would be the Deciduous (D) Low Canopy (L) Gleaning (G) Insectivores (I) that are associated with an edge patch type (2). This is abbreviated as DLGI2. (See Hamel et al. 1982)

b Species categorized as carnivores, plantivores, graminivores, or scavengers were not included in the final analysis due to insufficient sample sizes or the occurrence of extensive portions of territories of these generally wide-ranging species far beyond plot boundaries

OR 6. Assignment of 59 breeding bird species at the Connecticut Hill WMA to guilds using the classification system outlined in OR 5[a]. From Keller et al. (2003).

1) DLGI1	2) DMGI1	3) DHGI1	4) DTGI1	5) DUGI1	6) CHGI1	7) OTGI1
COYE[b]	BCCH	REVI[c]	SOSP	VEER	BTGW	GRSP
CSWA	CEWA[c]	SCTA	EATO	WOTH	BLBW	SASP
AMGO	RBGR		FISP	OVEN	BHVI	RWBL[c]
GRCA	BBCU		WTSP	AMRO[c]	MAWA	BOBO
CAWA	YBCU		NOFL[c]	HETH		EAME
MOWA	REVI[c]		RWBL[c]			
BAWW[c]			COGR			
RTHU[c]			EABL[d]			

8) CTGI2	9) DLGI2	10) DLSI2	11) MHSI2	12) DBGI2	13) MLGI2	14) DTGI2
CHSP	INBU	ALFL	EWPE	BAWW[c]	BTBW	AMRO[c]
DEJU	HOWR		GCFL			AMWO
	NAWA					
	BWWA					
	PRWA					
	NOCA					
	BRTH					
	GWWA					
	YBCH					

15) MMSI2	16) CHGI2	17) DBGI1	18) DBPI	19) DLHN2
CEWA[c]	PUFI	WBNU	HAWO	RTHU[c]
		BRCR	YBSA	
			DOWO	
			PIWO	
			NOFL[c]	

a Guilds listed include all species used in the final data analysis. Within guilds, species are listed in general order of decreasing relative abundance. Guilds were numbered to facilitate reference from the text. Numbers generally correspond to patch types identified in Table 1 and more fully described in OR 8

b Standardized abbreviations follow USGS Bird Banding Laboratory (BBL) Codes (https://www.pwrc.usgs.gov/bbl/MANUAL/speclist.cfm). See OR 4

c Species was placed in more than one guild based on observed foraging behavior and plant community structured

d Although the EABL uses a different foraging tactic (i.e., pouncing) than the other members of DTGI1, it was judged to consume essentially the same prey items

OR 7. Landscape component classification system for the Connecticut Hill WMA in Tompkins County, NY. Percentages are the proportion of a single GIS map cell (actual area = 100 m-2) represented by the type.

Landscape Component #	Description
1	>85% sprouts[a] or shrubs
2	10-85% sprouts or shrubs
3	10-85% sprout/sapling conifers
4	<10% sprouts or shrubs (i.e., open grass)
5	deciduous sapling/pole w/>33% sprouts
6	deciduous sapling/pole w/<33% sprouts and >67% canopy closure
7	deciduous sawtimber w/>33% sprouts
8	deciduous sawtimber w/<33% sprouts
9	conifer pole/sawtimber w/>33% sprouts or w/live branches to ground level
10	conifer pole/sawtimber w/<33% sprouts or w/dead branches to ground level
11	fruit trees or tall (>3 m) shrubs
12	bare ground
13	water
14	mixed deciduous-coniferous sapling/poletimber
15	mixed deciduous-coniferous sawtimber
16	deciduous sapling/poletimber w/<33% sprouts and <67% canopy closure

a Tree diameter classes:
Sprout: <4 cm dbh (included seedlings, root suckers, and stump sprouts)
Sapling: 4-10 cm dbh
Poletimber: 10-20 cm dbh
Sawtimber: >20 cm dbh

OR 8. Definitions of 16 exploratory patch types (T) using the landscape component classification system in OR 7. Patches 1-7 are solid types (see text). Patches 8-16 are edge types and are identified in the description by the '/' between adjacent patch types composing the edge.

Type T Number[a]	Patch Type (T) Description	Landscape components[b] composing the patch type	Vertical Profile[c]
1	Deciduous Dense Shrubs	1, 5, 7, 9 (w/deciduous understory)	0-3m
2	Deciduous Middle Canopy	1(>3m), 5, 6, 11	3-7m
3	Deciduous High Canopy	7, 8	>7m
4	Open Shrub	2, 3, 11, 16	0-3m
5	Deciduous Understory	1(>3m), 5, 6, 7, 8, 14, 15, 9 (w/ deciduous understory)	>3m
6	Mixed Deciduous-Coniferous Canopy	9, 10, 15	>7m in NHH
7	Open Grass	4	0-1m
8	Coniferous / Opening	2 2 2 3 3 4 4 9 10[d]<br>9 10 15 9 10 9 10 12 12	0-3m
9	Shrub / Opening	1 1 1 1 2 2 2 2 3 3 4 4 5<br>2 3 4 16 5 7 9 11 5 7 5 7 16	0-3m
10	Sapling / Opening	2 2 3 5[e] and +1 1[f] or 1[g]<br>5 11 5 11 5 11 2	0-3m
11	Interior Canopy / Opening[h]	1 1 2 2 2 2 3 3 5 5 5 5 6 6 7 7 8 8<br>7 8 7 8 14 15 7 8 7 8 14 15 7 8 11 16 11 16	>7m
12	Interior Canopy / Shrub[h]	1 1 5 5 7<br>7 8 7 8 8	0-3m
13	N Hardwood-Hemlock / Shrub	1 5 7<br>15 15 15	0-3m
14	Shrub-Sapling / Opening	1 1 1 1 1 2 2 2 2 2 2 2 2 4 4 4 4 4 4 5 5<br>2 3 4 16 13 3 4 5 6 7 11 13 16 5 6 7 11 16 13 16	0-3m
15	Interior Mid-canopy / Opening[h]	1 1 2 2 2 3 3 4 4 4 5 5 5 6 6 11 11<br>5 6 5 6 11 5 6 5 6 11 11 12 16 11 16 12 16	3-7m
16	Coniferous Canopy / Shrub	1 1 3 3 5 5<br>9 10 9 10 9 10	0-7m

a Numbering system follows Keller 1986 and 1990, and Keller and Smith 2014
b Numbers refer to landscape component numbers in OR 7
c Portion of vertical profile to which measurements of patch size and total amount applied for each patch type
d Each 2(row) x N(column) matrix represents all the combinations of adjacent landscape components (OR 7) composing an edge patch type. For example, 2 above 9 is open shrubs (type 2) adjacent to conifer pole/sawtimber w/>33% sprouts or live branches to ground level (type 9)
e Used for jack pine clearcuts (CCA-4,8,9) in 1977-78
f Used for all CCA sites other than jack pine clearcuts in 1977-78, for all CCC and OF sites in all years, and for CCA-4,8,9 in 1979-80

g Used for all CCA sites in 1979-81 except CCA-4,8,9 and for both CCB sites in all years
h Measurements of the patch type (e.g., using ESCAN) were made only on internal edges (i.e., not including the site border)

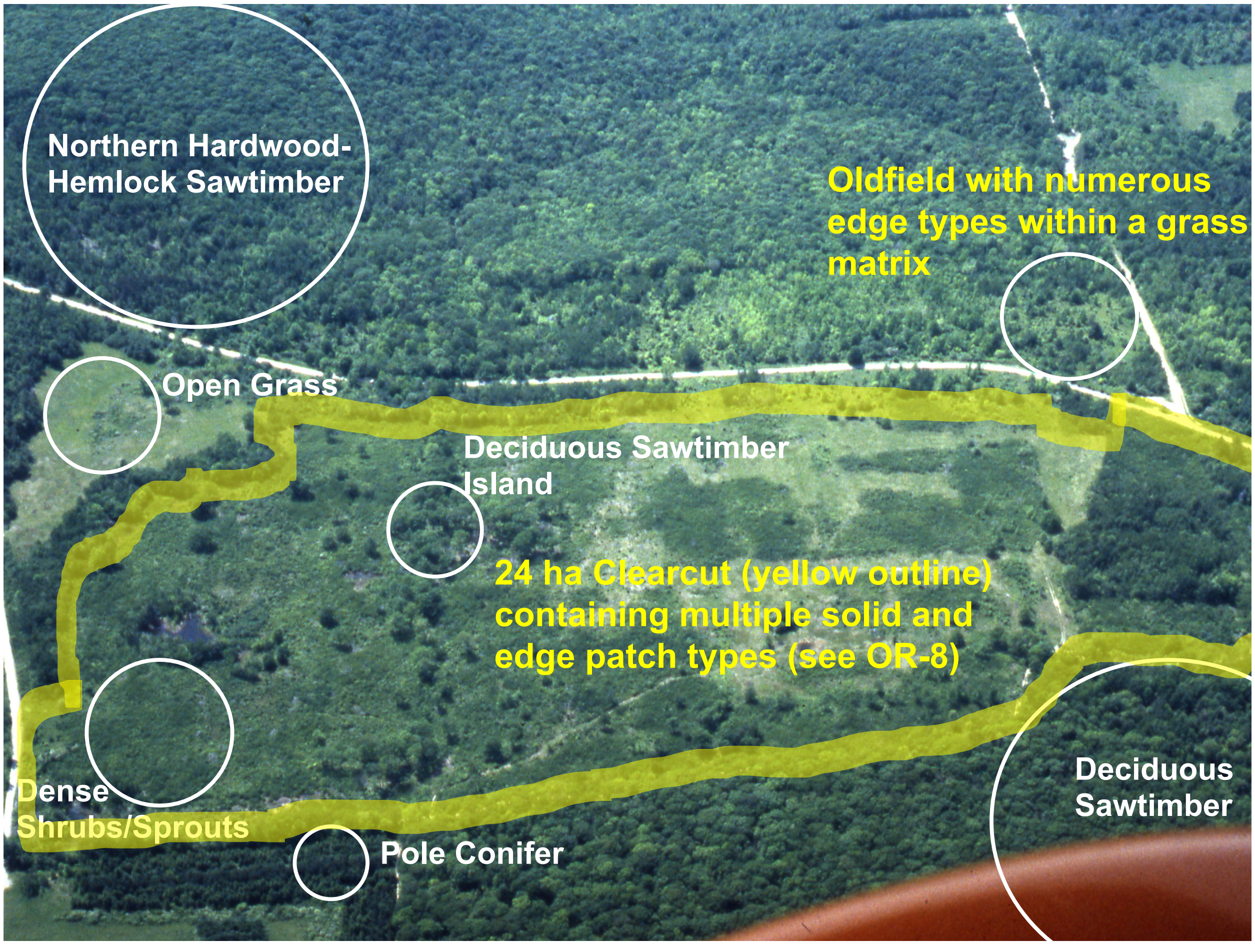

OR 9 MDC's for different solid patch types within broader cover types of forest and clearcut. Note the heterogeneity and resulting high number of edges (e.g., trees adjacent to open grass) within the oldfield at upper right. This is inconsistent with the concept of MDC as pertaining to homogeneous patch types, thus leading to the desirability of a comparable variable (DEAC) to quantify the density of edge types associated with particular species or guilds. Also note the many types of edges that occur within the more general classification of the oldfield and 24 ha clearcut outlined in yellow (see Fig. 4 for examples of edge types at higher spatial resolution)

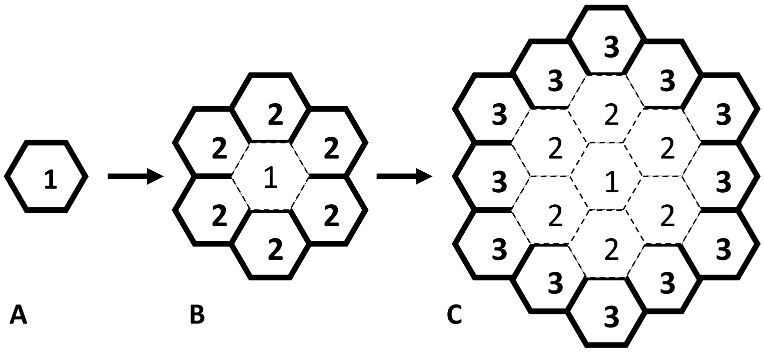

OR 10 The pattern of edge addition (solid lines) to the progressively larger samples examined by the edge scanning algorithm ESCAN. A prespecified number of annuli are examined for the presence of type T edges starting within each cell on the GIS map. See OR 11 for an example of the output from this program. To see how this approach can be applied as a practical measure of habitat size and quality for edge-associated species, examine the "Poor" vs "Good" cover type interspersion of northern bobwhite quail (*Colinus virginianus*) habitat quality depicted in fig. 9 of Leopold (1933). **A** 1 annulus sample = 6 total edges, **B** 2 annuli sample = 30 total edges, **C** 3 annuli sample = 72 total edges. From Keller and Smith 2014

OR 11. An example of the output from the edge density scanning algorithm ESCAN (OR 10) as applied to shrub-sapling / opening edge within a hypothetical oldfield (Fig. 5). The program locates the area on the map with the highest density of edges of any specified patch type (T) for each sample size (i.e., number of annuli, column 1), then compares edge density across all sample sizes and selects that sample size with the highest absolute edge density (EI), in this case EI=9.7. The area of cells within the number of annuli exhibiting the highest value of EI is converted to DEAC (last column), the diameter of the equivalent area circle. See Figure 5 and the text for further description of the technique. From Keller and Smith (2014).

Annulus[a] Size	Starting Cell[b] I	J	# Type T	Total Edges	% T	AAS[c]	EI[d]	DEAC[e]
1	2	8	6	6	1.000	1	6.00	
2	3	7	18	30	.600	7	6.80	
3	4	5	43	72	.597	19	9.70	49.19
4	4	5	70	129	.543	37	9.54	

a Number of annuli (i.e., rings of cells) in the sample
b Location of the row (I) and column (J) of the sample center on the hexagonal-celled map depicted in Figure 5
c The Actual Area (# of cells) Sampled by the procedure
d Modification of Patton's Edge Index: EI = No. of type T edges in the sample $AAS^{-.5}$
e Diameter of the Equivalent Area Circle (m); calculated only for the annulus size >1 with the highest EI

OR 12

Rank sum test used to identify nonrandom patterns of species additions to a guild based on body size. Guild members are ranked 1-i, from smallest to largest. From Keller 1986.

$$Z = \frac{\bar{r} - \frac{G+1}{2}}{\sqrt{\frac{1}{N}\left(\frac{1}{K} - \frac{1}{G}\right) - \frac{G(G+1)}{12}}}$$

where

$\bar{r}$ = Rank Mean = $\frac{\sum_{i=1}^{N} (S_i \Sigma R_i)}{KN}$

R_i = rank of species i
S_i = # of observations of species or species combination i

K = # of species actually present (i.e., observed) in the guild
G = Total # of species in the guild
N = Total # of observations of guild size K

Testing for the nonrandom occurrence of the initial guild member based on body size:

Theoretically, small species are more likely to be the first species to occur in smaller patches because they require less patch area in which to breed (Schoener 1983). This leads to small species generally being more abundant than larger species. A possible null hypothesis based on observed frequency of occurrence would include, and therefore obscure, this potential effect of body size (Colwell and Winkler 1984). Therefore, our null hypothesis was that all species had an equal likelihood of occurrence. Except for the mid-canopy foliage-gleaners (Guild 2), which were of interest because of the wide range of distinct body sizes they presented, no attempt was made statistically to analyze higher orders of species combinations within guilds (Keller 1986).

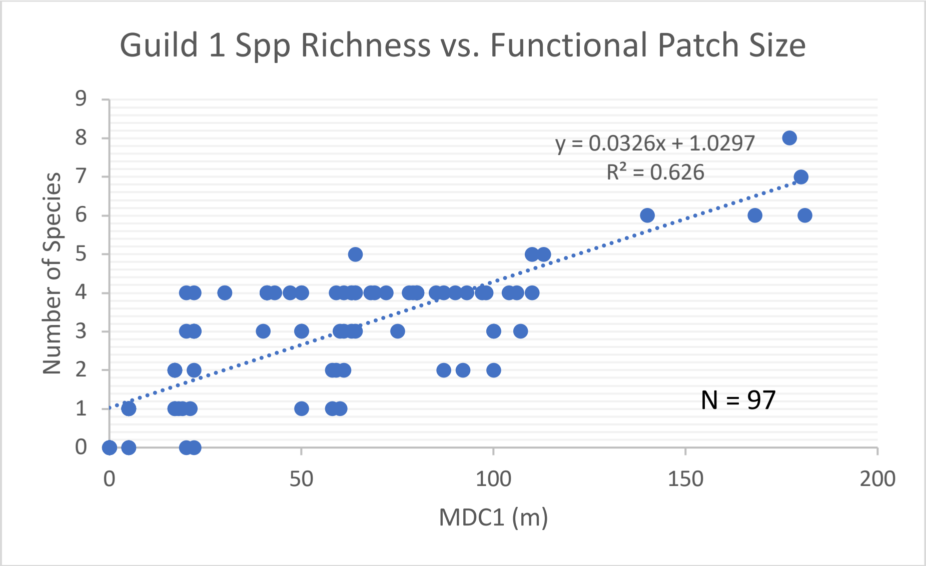

OR 13 Linear regression model of Guild 1 species richness with functional patch size (m). See Table 3

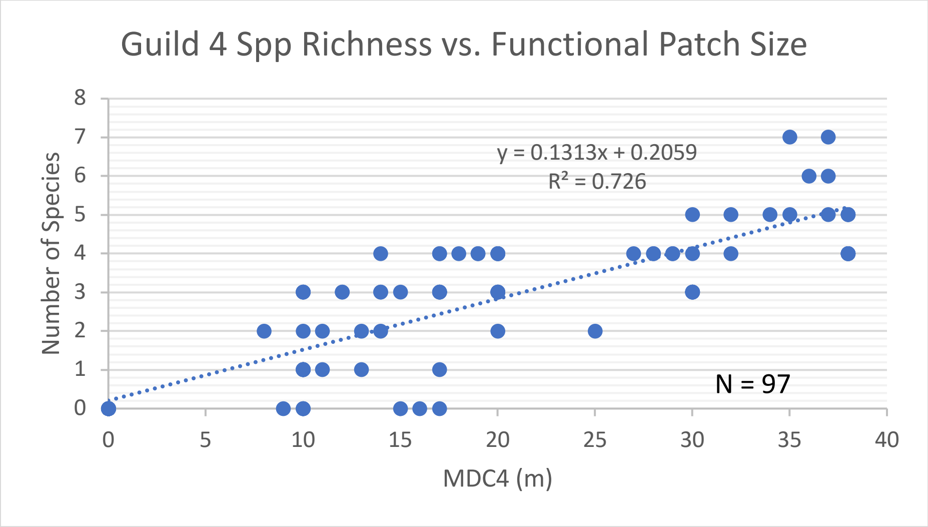

OR 14a Linear regression model of Guild 4 species richness with functional patch size (m)[2]. MDC4 was the primary variable in the most parsimonious multivariate regression model for Guild 4, the terrestrial gleaning insectivores (Table 3)

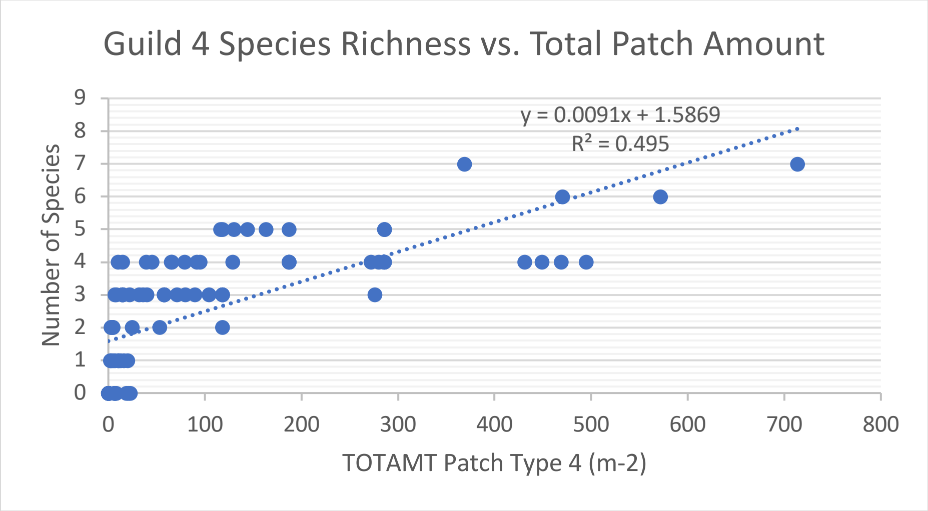

OR 14b Linear regression model of Guild 4 (terrestrial gleaning insectivores) species richness with Total Patch Amount (m-2). Based on AIC values and the Cp statistic, the multivariate model including TOTAMT4 was not plausibly equivalent to the most parsimonious model for species richness of Guild 4 (Table 3)

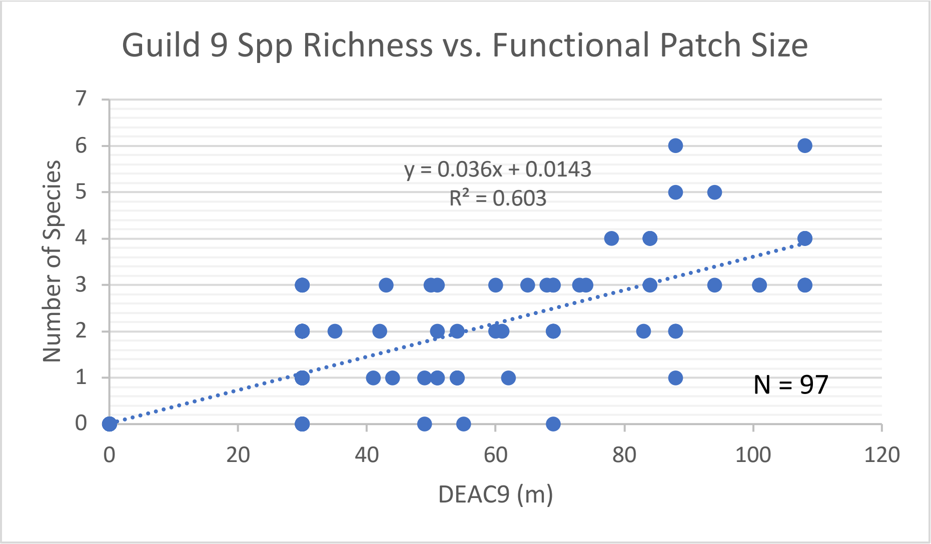

OR 15a Linear regression model of Guild 9 species richness with functional patch size (m). DEAC9 was the sole variable in one of the two most plausible multivariate regression models (Table 3) for Guild 9, the deciduous low-canopy-edge gleaning insectivores (DLGI2, OR 5 and OR 6)

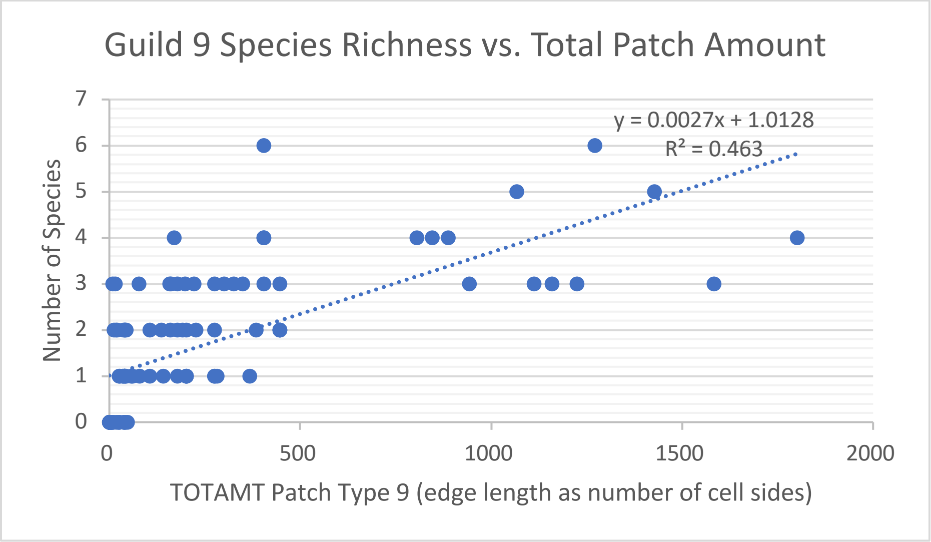

OR 15b Linear regression model of Guild 9 species richness with Total Patch Amount. TOTAMT9 was a secondary variable in one of the two most plausible multivariate regression models (Table 3) for Guild 9, the deciduous low-canopy-edge gleaning insectivores (DLGI2, OR 5, OR 6)

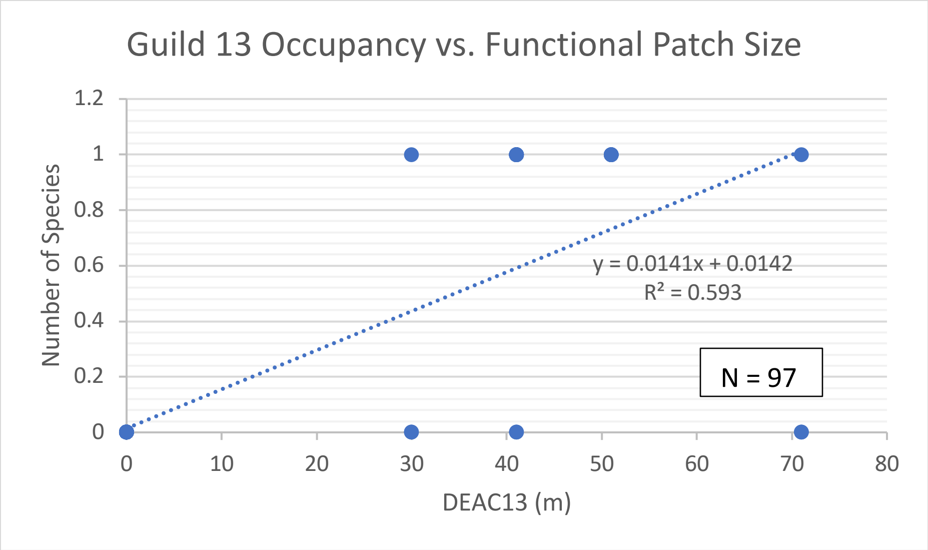

OR 16a Linear regression model of Guild 13 (black-throated blue warbler *Setophaga caerulescens*) occupancy with functional patch size (m) (MLGI2, OR 5 and OR 6). Like TOTAMT below, although highly significant, DEAC13 was not the strongest predictor of patch occupancy for this guild.

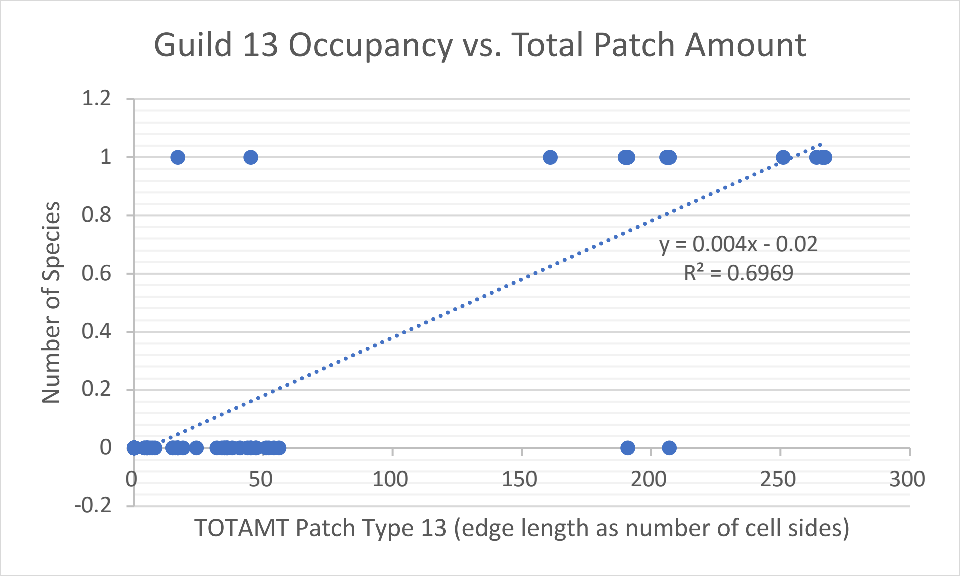

OR 16b Linear regression model of Guild 13 occupancy with TOTAMT13. Although TOTAMT13 was a highly significant predictor of occupancy for Guild 13 (black-throated blue warbler *Setophaga caerulescens* (MLGI2, OR 5 and OR 6), within-patch heterogeneity (NUMHAB13) was a better predictor ($R^2 = 0.721$) in the multivariate regression model (OR 16c, Table 3)

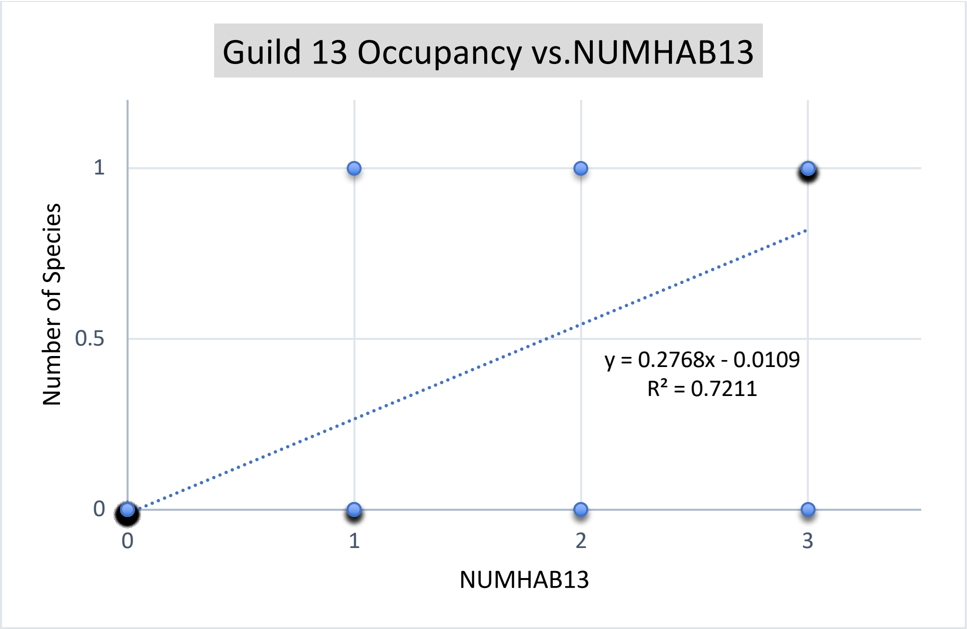

OR 16c Linear regression model of Guild 13 occupancy with the within-patch heterogeneity (NUMHAB) of Patch Type 13 present on a site. NUMHAB13 was the best predictor of occupancy for Guild 13 (black-throated blue warbler *Setophaga caerulescens* (MLGI2, OR 5 and OR 6, Table 3)

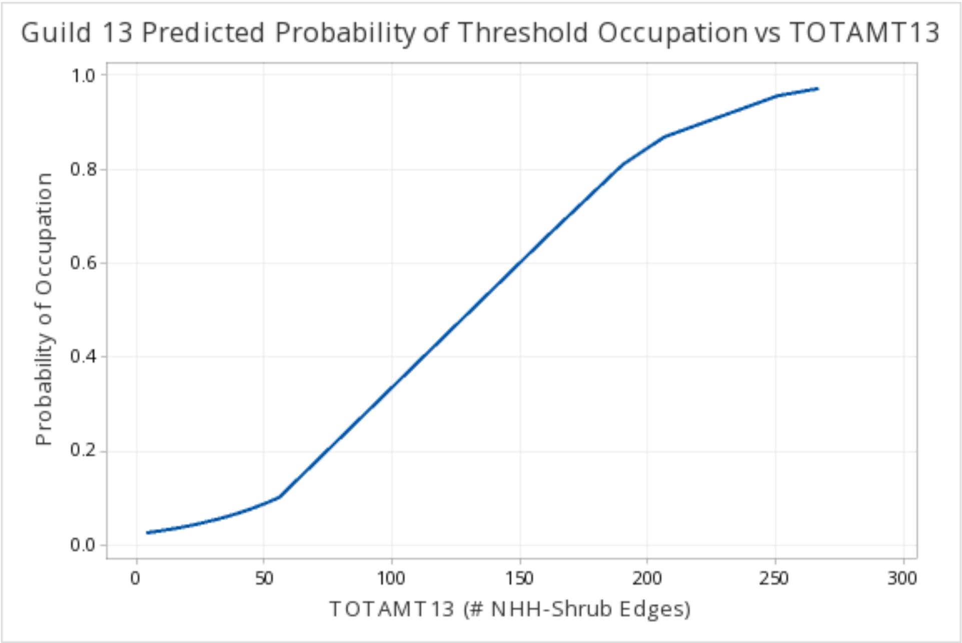

OR 16d Predicted probability of threshold occupation of the black-throated blue warbler *Setophaga caerulescens* (Guild 13, MLGI2) vs. total amount of Patch Type 13 (northern hardwoods-hemlock/shrub edge). Probability of occupation is near 0 at small amounts of the edge type and increases to near 1.0 as TOTAMT13 approaches 1700 m (275 edgelengths x 6.2 m per cell side) on a site. Total amount of patch Type 13 edge was significantly greater ($\bar{x}_{TOTAMT}$ = 191 edges = 1184 m) on occupied plots than on unoccupied plots ($\bar{x}_{TOTAMT}$ = 36 edges = 223 m, t-test, $p<0.001$, N=79)

OR 17. Effect Tests (F Ratio) for predictor variables when multiple variables were included in plausible (AIC) linear regression models of avian within-guild species richness at the Connecticut Hill WMA in central New York (Table 3). Predictor variables entered in trials for each guild are those identified in Table 3. Primary variable in the model is in bold.

Guild	Model R-SQ	Predictor Variables	F Ratio	Prob > F	% of Explained Variance
1) DLGI1 [a]	0.647*** [b, c]	**MDC1** [d]	56.7	<0.0001	92
		NUMHAB1	5.19	0.0251	8
2) DMGI	0.391***	**MDC2**	58.627	<0.0001	77
		NUMHAB2	27.975	<0.0001	23
	0.410***	**TOTAMT2**	60.1	<0.0001	60
		NUMHAB2	25.4	<0.0001	25
		SITESIZE	15.4	0.00017	15
3) DHGI!	0.841***	**MDC3**	448	<0.0001	99
		SITESIZE	4	0.0475	1
4) DTGI1	0.740***	**MDC4**	237.8	<0.0001	98
		SITESIZE	5.07	0.027	2
5) DUGI1	0.721***	**MDC5**	233.05	<0.0001	98
		NUMHAB5	4.302	0.0481	2
9) DLGI2	0.615***	**NUMHAB9**	37.02	<0.0001	63
		TOTAMT9	22.01	<0.0001	37
10) DLSI2	0.352***	**DEACA10**	13.719	0.0004	62
		NUMHAB10	8.568	0.0043	38
11) MHSI2	0.511***	**DEACA11**	43.967	<0.0001	81
		SNAGTOT	10.325	0.0019	19
12) DBGI2	0.380***	**SITESIZE**	13.792	0.00035	38
		TOTAMT12	13.51	0.0004	38
		NUMHAB12	8.596	0.00424	24
14) DTGI2	0.718***	**NUMHAB14**	32.419	<0.0001	42
		SITESIZE	25.663	<0.0001	33
		TOTAMT14	19.899	<0.0001	25
16) CHGI2	0.304***	**DEACA16**	37.016	<0.0001	84
		SITESIZE	7.184	0.0087	16
18) DBPI	0.727***	**SNAGTOT**	27.15	<0.0001	67
		TOTAMT3	13.154	0.00047	33

a Guild classification and composition in OR 5 and OR 6, respectively

b ** *P*<0.01, *** *P*<0.0001

c All N = 97

d Variable definitions in Table 2. Variables listed in order of decreasing t Statistic value in the model

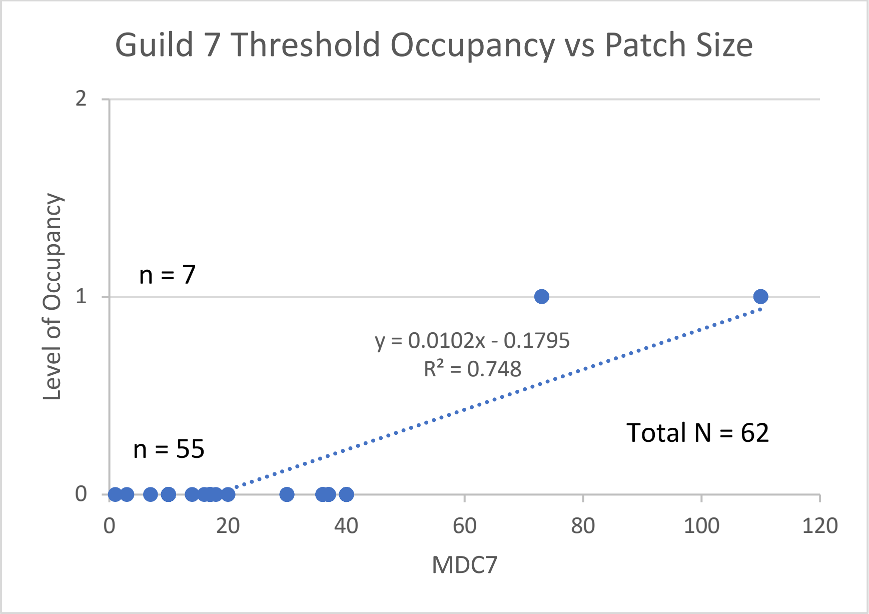

OR 18 Categorical linear regression model illustrating threshold occupancy of patches of Type 7 (open grass) by breeding birds of Guild 7 (OTGI1). Distribution of functional patch sizes (MDC [m]) is shown at 2 occupancy levels - unoccupied (0) and occupied but not including the largest species (1). Occupied patches were significantly larger ($\overline{x}_{MDC} = 94$ m) than unoccupied patches ($\overline{x}_{MDC} = 20$ m). See Table 4 and OR 19

OR 19. Average size (MDC or DEAC) of patch type T[a] for unoccupied patches of type T vs. the average size of type T patches with occurrence of the guild in the absence of the largest species, and with occurrence of the largest species in each of the 13 multispecies guilds. Statistical differences between the categories are noted. For guild abbreviations and guild members, see OR 5 and OR 6.

		$\bar{x}$ MDC or DEAC (m)			
Guild[a]	Largest Species	A<br>Unoccupied<br>Type T Patches	B<br>Guild w/o<br>Largest Species	C<br>Guild w/<br>Largest Species	t test
1) DLGI1 (8[b])	GRCA	11 (5)	40 (28)	74 (55)[c]	*ab*, **ac**, **bc**[d]
2) DMGI (6)	BBCU, YBCU	36 (26)	94 (57)<br>67 (7)[e]	122 (14)<br>116 (9)[e]	**ab**, **ac**, bc+<br>**ab**, **ac**, **bc**
3) DHGI1 (2)	SCTA	14 (51)	30 (10)	169 (20)	**ab**, **ac**, **bc**
4) DTGI1 (7)	NOFL, COGR	12 (7)	19 (55)	32 (17)	*ab*, **ac**, **bc**
5) DUGI1 (5)	WOTH	25 (31)	73 (41)	159 (25)	**ab**, **ac**, **bc**
6) CHGI1 (4)	BHVI	17 (54)	124 (8)	180 (8)	**ab**, **ac**, *bc*
7) OTGI1 (5)	EAME	20 (55)	92 (7)	73 (1)	**ab**, ac IS, bc IS
8) CTGI2 (2)	DEJU	28 (29)	46 (33)	41 (20)	**ab**, **ac**, bc NS
9) DLGI2 (9)	BRTH, NOCA, YBCH	38 (10)	57 (58)	79 (11)	*ab*, **ac**, **bc**
11) MHSI2 (2)	GCFL	50 (43)	90 (11)	76 (7)	**ab**, **ac**, bc NS
14) DTGI2 (2)	AMWO	32 (16)	57 (23)	71 (40)	**ab**, **ac**, *bc*
17) DBGI1 (2)	WBNU	26 (66)	180 (1)	180 (14)	ab IS, **ac**, bc IS
18) DBPI (5)	PIWO	19 (55)	116 (18)	160 (8)	**ab**, **ac**, bc NS
**$\bar{x}$ All Guilds**		**<u>25</u>** (448)	**62** (350)	**<u>100</u>** (240)	

a For patch type T – guild associations see Table 1
b Numbers in parentheses in this column are the number of species that could occur in the guild
c Numbers in parentheses in columns A, B, and C are the number of samples (N) of site survey years in the category
d t test
ab $\bar{x}$ patch size of unoccupied patches vs. $\bar{x}$ patch size with guild present without largest species
ac $\bar{x}$ patch size of unoccupied patches vs. $\bar{x}$ patch size with largest species
bc $\bar{x}$ patch size with guild present without largest species vs. $\bar{x}$ patch size with largest species
+ p=0.06, *p<0.03 (Italics)*, **p<0.001 (BOLD),** NS Not Significant, IS Insufficient samples to test statistically
e Clearcuts CCA and CCB 1980-81 only; Rank Sum Test

OR 20. Predicted threshold and effect sizes of functional patch size (MDC/DEAC) in linear regression models of species richness within multispecies avian guilds at the Connecticut Hill WMA in central New York.

Guild	Variable R-SQ	Predictor Variable	Predicted Threshold Size	Effect Size for each Additional Species
1) DLGI1 (6) [a,b]	0.623*** [c,d]	**MDC1**[e]	<5m	+30m
2) DMGI (6)	0.210*	**MDC2**	29 m	+95 m
3) DHGI1 (2)	0.835***	**MDC3**	89 m	+89 m
4) DTGI1 (7)	0.726***	**MDC4**	6 m	+7 m
5) DUGI1 (5)	0.709***	**MDC5**	59 m	+51 m
6) CHGI1 (4)	0.836***	**MDC6**	120 m	+120 m
7) OTGI1 (5)	0.615***	**MDC7**	70 m	+70 m
8) CTGI2 (2)	0.307**	**DEAC8**	47 m	+38 m
9) DLGI2 (9)	0.603***	**DEAC9**	27 m	+28 m
11) MHSI2 (2)	0.448**	**DEAC11**	110 m	+113 m
14) DTGI2 (2)	0.638***	**DEAC14**	46 m	+45 m
17) DBGI1 (2)	0.712***	**MDCA3**	162 m	+160 m
18) DBPI (5)	0.621***	**MDCA3**	119 m	+127 m

a Guild classification and composition in OR 5 and OR 6, respectively
b Number in parentheses is the maximum number of species in the guild ever observed on any site
c * p<0.05, ** p<0.01, *** p<0.0001
d All N = 97 except Guilds 8 and 11 (N=84)
e Variable definitions in Table 2

OR 21. Trends in the occurrence of the initial species, based on body weight, for the 13 multispecies guilds. Those guilds for which the occurrence of the smallest species or species combination is significantly nonrandom are noted. For guild abbreviations and guild members, see OR 5 and OR 6.

Guild	# of Species Observed--	Total Samples	Species Observed (# observations)	p^b
1) DLGI1 (6[a])	1	N=14	COYE (n=8)<br>CAWA (n=6)	**
2) DMGI (6)	1	N=20	BCCH (n=12)<br>CEWA (n=6)<br>RBGR (n=2)	***
	2	N=13	BCCH, CEWA (n=5)<br>BCCH, RBGR (n=5)<br>CEWA, RBGR (n=3)	***
	3	N=10	BCCH, CEWA, RBGR (n=5)<br>BCCH, CEWA, BBCU (n=2)<br>BCCH, RBGR, YBCU (n=2)<br>BCCH, RBGR, BBCU (n=1)	***
3) DHGI1 (2)	1	N=10	REVI (n=10)	***
4) DTGI1 (7)	1	N=8	EATO (n=8)	NS
5) DUGI1 (5)	1	N=32	VEER (n=30)<br>OVEN (n=1)<br>WOTH (n=1)	NS
6) CHGI1 (4)	1	N=6	BTGW (n=3)<br>MAWA (n=2)<br>BLBW (n=1)	*

[a] Number in parentheses is the number of species that could occur in the guild. For Guild 1 (DLGI1), the ruby-throated hummingbird and black-and-white warbler were not included in this analysis because they were classified as foliage-gleaners for only 4 of 28 and 5 of 15 observations, respectively. Those observations included at least 4 other guild members

[b] Rank Sum Test (see OR 12)
* $P < 0.05$, ** $P < 0.01$, *** $P < 0.001$, NS Not Significant

OR 21 (continued)

Guild	# of Species Observed--	Total Samples	Species Observed (# observations)	p^b
7) OTGI1 (5)	1	N=4	GRSP (n=4)	**
8) CTGI2 (2)	1	N=49	CHSP (n=30)<br>DEJU (n=19)	0.058 NS
9) DLGI2 (9)	1	N=20	INBU (n=18)<br>HOWR (n=1)<br>NAWA (n=1)	NS
11) MHSI2 (2)	1	N=24	EWPE (n=17)<br>GCFL (n=7)	*
14) DTGI2 (2)	1	N=25	AMRO (n=23)<br>AMWO (n=2)	***
17) DBGI1 (2)	1	N=10	WBNU (n=8)<br>BRCR (n=2)	NS
18) DBPI (5)	1	N=8	HAWO (n=3)<br>YBSA (n=2)<br>DOWO (n=1)<br>PIWO (n=1)	NS

[a] Number in parentheses is the number of species that could occur in the guild. For Guild 1 (DLGI1), the ruby-throated hummingbird and black-and-white warbler were not included in this analysis because they were classified as foliage-gleaners for only 4 of 28 and 5 of 15 observations, respectively. Those observations included at least 4 other guild members

[b] Rank Sum Test (OR 12)

* $P < 0.05$, ** $P < 0.01$, *** $P < 0.001$, NS Not Significant

OR 22. Simple linear correlations (r) of guild species richness of breeding birds with measures of functional patch size and total patch amount of guild specific patch types on 23 clearcuts, oldfields, and forests surveyed between 1977 and 1981 at the Connecticut Hill WMA in central New York. Guild species richness-patch metric correlations attempted match patch type associations identified in Table 1. Values in **bold** indicate highest correlation for the guild. All highest correlations were highly significant (p<0.001) except for Guild 19 (ruby-throated hummingbird), p<0.01.

Guild	1	2	3	4	5	6	7
Variable	DLGI1[a]	DMGI1	DHGI1	DTGI1	DUGI1	CHGI1	OTGI1
MDC / DEAC[b]	**0.791**[cd]	0.458	**0.914**	**0.852**	**0.842**	0.910	0.715
MDCA / DEACA	0.690	0.368	0.891	0.785	0.811	**0.914**	**0.784**
SITESIZE	0.128	0.155	0.248	0.586	0.172	0.031	-0.023
TOTAMT	0.529	**0.482**	0.878	0.703	0.741	0.895	0.654
SNAGTOT		0.186					

Guild	8	9	10	11	12	13
Variable	CTGI2	DLGI2	DLSI2	MHSI2	DBGI2	MLGI2
MDC / DEAC	**0.551**	**0.776**	**0.523**	0.610	0.291	0.770
MDCA / DEACA	0.546	0.698	0.511	**0.670**	0.187	0.657
SITESIZE	0.027	0.543	0.391	0.354	0.027	0.138
TOTAMT	0.343	0.681	0.516	0.543	**0.497**	**0.835**
SNAGTOT				0.088		

Guild	14	15	16	17	18	19
Variable	DTGI2	MMGI2	CHGI2	DBGI1	DBPI	DLHN2
MDC / DEAC	**0.799**	**0.862**	0.413	0.844	0.772	0.282
MDCA / DEACA	0.699	0.801	**0.487**	**0.865**	0.788	0.306
SITESIZE	0.372	0.260#	0.204	0.144	0.112	0.228
TOTAMT	0.619	0.685	0.401	0.843	0.805	**0.313**
SNAGTOT				0.766	**0.830**	

a Guild abbreviations as in OR 5.
b Variable definitions are found in Table 2.
c All N = 97 except Guilds 8 and 11 (N=84) and Guild 15 (N=45)
d Value in **bold** indicates highest correlation for the guild.
Values <0.199 = Not Significant (NS)
Values >0.199<0.26 = p<0.05
Values >0.26<0.33 = p<0.01
Values >0.33 = p<0.001
NS based on smaller sample size (N=45) for this guild

OR 23. Linear regression models of Total Species Richness (SR), Guild Richness, and Shrubland Bird SR with SITESIZE (Table 2).

Variable	Model R-SQ
Total SR	0.362[a]
# Guilds	0.157
Shrubland Bird SR	0.403

All P<0.0001, N=97 for Total SR and # Guilds, N=79 For Shrubland Bird SR

OR 24. Linear regression models of species richness of the 13 multispecies guilds with the internal heterogeneity (number of landscape components, OR 8) of their associated patch types (the variable NUMHAB, Table 2) actually present on each site. For guild abbreviations and guild members, see OR 5 and OR 6.

Guild[a]	NUMHAB
1) DLGI1	0.435***
2) DMGI	0.011 NS
3) DHGI1	0.224***
4) DTGI1	0.462***
5) DUGI1	0.030 NS
6) CHGI1	0.008 NS
7) OTGI1	0.040*
8) CTGI2	0.039 NS
9) DLGI2	0.525***
11) MHSI2	0.019 NS
14) DTGI2	0.638***
17) DBGI1	0.125**
18) DBPI	0.163***

a For patch type T–guild associations see Table 1
b * p<0.05, ** p<0.001, ***p<0.0001, NS Not Significant

Online Resource 25 – Refining Guild-Patch Associations

Prediction Inconsistencies

The potential for climatic or other environmental uncertainty to disrupt the influence of factors such as habitat structure, which may serve to organize communities, has been well documented by Paine (1966, 1984), Wiens (1977), and others. In this study, fluctuations in the richness of resident species, the apparently random composition of single-species occurrences of resident woodpeckers (Guild 18, Table 5) and bark-gleaners (Guild 17), and absences of the black-capped chickadee as the initial representative of its guild (DMGI1, Guild 2, Table 5) may be at least partially attributable to the negative effects of severe overwintering conditions on survivorship of the smallest members of these guilds (Keller 1986, Root 1988; Meehan et al. 2004). As a result, suitable patches for such residents may remain unsaturated because, even when individual habitat requirements are met, overwintering conditions can reduce population levels of some or all guild members (cf. O'Conner and Fuller 1983).

Patch conditions related to a species' natural history traits (e.g., lack of appropriate nest sites [Scott 1979; Dickson et al. 1983]) also may influence the sequence of species appearance in a guild. Snag densities were significantly lower on 6 of 7 plots where the chickadee was not the first species of its guild to appear. Natural history traits of the resident bark gleaners (DBGI, Guild 17) also may help explain the occurrence of the larger white-breasted nuthatch rather than the brown creeper as the more frequent representative in single-species occurrences of this guild (Table 5). The creeper is more restricted to mature forests than the nuthatch and has more restrictive nest site requirements (beneath loose bark as opposed to secondary cavities) (Hejl et al. 2002; Pravosudov and Grubb 1993).

Stronger Correlations of SR with Total Patch Amount

Only 5 of the 19 guilds had stronger relationships of species richness with total patch amount than with functional patch size (OR 21). Of these 5, the black-and-white warbler and black-throated blue warbler appear to be forest-interior-edge (canopy gap) adapted (Goetz et al. 2010; Keller and Smith 2014). Thus, they may only require a small amount (i.e., minimal DEACs) of their preferred edge type to establish territories and may tend to avoid larger openings. More detailed research on within-territory habitat structure, particularly for the black-and-white warbler, might better illuminate its habitat association with the edge between mature trees and regenerating forest or dense understory (Yahner 1986). Ultimately all of the first-cut species- / guild-habitat associations can be refined further. Lastly, the ruby-throated hummingbird also maintains a very small defended territory (Robinson, et al. 1996). However, the hummingbird is much less conspicuous than syntopic passerines detected in the study and as a result, its observed distribution may include sampling bias.

The difficulty of identifying patch types that are internally uniform across landscapes

The veery (Guild 5) provides an example of the difficulty, even when employing HR imagery, of identifying patch types that are internally uniform enough that associated individual species habitats are evenly represented across the full range of within-patch heterogeneity. First, although the veery is larger than the ovenbird, the smallest species in the guild, its territory size is reportedly smaller than that of the ovenbird (Van Horn and Donovan 1994; Bevier et al. 2004; Derochers et al. 2010, table 1). Thus, by a territory size definition of expected occurrence, the veery would be expected to occur more often as sole representative of the understory-gleaning guild. However, the veery's frequent appearance as the only member of the guild seems more related to its habitat structure than its smaller territory size.

Although the veery occurs in mature forests (Bertin 1977; DeGraaf and Rudis 1986), its association with dense understory coupled with a minimum requisite canopy height of only 2.5 m (Keller et al. 2003) broadens the range of forest age classes (cover types) in which it occurs. Dense understory was most prevalent on early successional clearcuts (CCA, CCB, N = 52 of 97 survey years) during post-cut years 4-10 where the veery became the most

abundant breeding bird by post-cut year 8 (Keller et al. 2003:552, table 5, fig. 6). None of the other members of this guild occurred on these early stage clearcuts leading to the overwhelming occurrence of the veery in single species incidences of the guild (30 of 32 single species occurrences, Table 5), despite it not being the smallest species in the guild.

Conversely, the ovenbird, predicted by size to be the first species of the guild to appear at threshold patch sizes, inhabits the very open shrub layer or understory typical of pole stage or older closed canopy forests (DeGraaf and Rudis 1986; Keller et al. 2003). As a result, the ovenbird occurred on no sites in the study younger than post-cut year 24 (Keller et al. 2003:552). Sites older than this (CCC-2, F-SGA, F-NHHB-D, Table A1) typically supported multiple members of the deciduous understory gleaners (Keller et al. 2003, table 5, fig. 7). Thus although "forest understory" represents a restrictive subset of "forest"-related patch types, its structure varies over a wide range of forest age classes. Within the patch type, the veery's habitat type extends across most of this successional breadth, while other members of the guild are restricted to smaller portions of the same successional gradient.

Online Resource 26 - Considering Within-Patch Heterogeneity: Why Spatial Resolution Matters

The occurrence of multiple guild-specific patch types within most local landscapes in this study (e.g., OR-9) and the significant relationship of species richness with intra-patch heterogeneity for 8 of 13 guilds (OR-24) support the observation that patch types defined at any scale tend to occur in clumped distributions or along gradients (Margules and Stein 1989); and so, too, do associated species distributions (May et al. 2019). As noted, this means that interpretations of the influence of patch size, total amount, and spatial arrangement on species richness rely heavily on how precisely species groups are defined, and on the composition and uniformity of associated patch types (Fahrig 2013:1656; MacDonald et al. 2021:13). Results here and elsewhere support the argument that species-habitat associations are the most appropriate way to determine delineation of patches (Kupfer et al. 2006; Shriner et al. 2006; Arponen et al. 2012; Haddad et al. 2017). If so, the *resolution* at which patch types are defined needs to approach the *grain* at which focal species or species groups perceive and respond to the subset of the landscape they occupy (Keller and Smith 2014). Otherwise, the inability to separate the subset of the landscape used by focal species from more general cover types dilutes the strength of species-habitat correlations (Huston 2002; Betts et al. 2014, fig.1; Gaston et al. 2017). In GIS-based analyses, this capability depends upon initial researcher choices of image spatial resolution, classification system, and minimum size delineation or minimum mapping unit (MMU) (Stohlgren et al. 1997; Wulder et al. 2004; Gallant 2009; McDermid et al. 2009; Keller and Smith 2014).

Most tests of the HAH have defined species groups and habitat patch types broadly (Fahrig 2013, fig. 1b). This level of classification appears most often associated with use of Landsat imagery in GIS-based analyses. Landsat has spatial resolution (GRD) ≥ 30 m. Even with a more restrictive classification such as "native forest" (Fahrig 2013, fig. 1bii), Landsat and other lower resolution (LR) imagery cannot resolve individual landscape elements such as trees and shrubs, or consistently correctly classify variously aged forest or agricultural types (Stoms 1992; Smith et al. 2003; Luoto et al. 2004; McDermid et al. 2009; Arponen et al. 2012; but see Shirley et al. 2013 use of unclassified Landsat imagery). This has several consequences.

First, it can result in larger minimum mapping units that often exceed the size of the home ranges of focal species, rendering impossible quantification of within-territory composition or structure that might explain a species presence in one patch but not another (Holland et al. 2004; Farrell et al. 2013; Keller and Smith 2014; Bombi et al. 2018; Rechsteiner et al. 2019). Second, the inability of LR imagery to accurately classify more restrictively defined (i.e., more uniform and potentially more ecologically appropriate) patch types means that individual patches of more broadly defined types (i.e., biotopes) are much more likely to contain multiple (guild-specific) patch types classified as a single cover type (Stohlgren et al. 1997; Bar-Massada et al. 2012; Keller and Smith 2014; compare OR 1, OR 2 and OR 9). This confounds interpretation of the individual effects of either patch size or total patch amount (Fletcher et al 2018). Note, although potentially cost prohibitive or simply unavailable until recently, free or low-cost HR imagery (e.g., Maxar Technology imagery embedded in Google Earth) is becoming increasingly available

worldwide for use with species or species groups that warrant HR analysis (Keller and Smith 2014, Rechsteiner et al. 2017).

The local scale relationship of species richness with structural heterogeneity (OR8, OR 24) points to 1) a more general relationship of richness with increasing environmental heterogeneity over larger geographic areas (Wiens 1989; Ricklefs and Lovette 1999; Tews et al. 2004; Kallimanis et al. 2008; MacDonald et al. 2018) and 2) the likelihood that at LR a smaller proportion of the total patch area classified as habitat actually represents habitat as perceived by the focal species or group (Goetz et al. 2010; Tattoni et al. 2012; Farrell et al. 2013; Betts et al. 2014, fig.1; Bombi et al. 2018). This is especially true for habitat specialists (Holland et al. 2005; Mathews et al. 2014; Rechsteiner et al. 2019).

Ultimately, LR classification limitations dictate defining species groups broadly to match the limit of patch type identification. Yet, equating broad cover types such as "forest" with "habitat" for all forest dwelling species, is an acknowledged oversimplification (Watling et al. 2020:7). This not only leads to the potential inclusion of non-habitat within the patch (type T) as noted above, but also raises the question of whether habitat breadth of some species may include other patch types that exist in the surrounding matrix (Andren et al. 1997)? Fahrig (2013:1656) recognized this problem and noted "To test the HAH directly, we need a single value of habitat amount for each sample site; it is not clear what that value would be if the habitat amount available to each species (both present and absent) is different."

As examples, roughly half of the Atlantic Forest small mammals identified to species and classified as "savanna woodland" species in Melo et al. (2017) or "forest" species in Vieira et al. (2018), are listed on the IUCN Red List of Threatened Species (iucnredlist.org/species/#habitat-ecology, 2016 assessments) as also occurring in other biotopes (e.g., shrubland, savanna, grassland, artificial [i.e., anthropogenic or degraded former forest]), which are acknowledged as potentially occurring in the surrounding matrices in both studies. Thus, for any species for which habitat associations are broader than "woodland" or "forest", interpretations of the influence of patch size or total amount would be less meaningful because, rather than entirely a barrier, the surrounding matrix may serve as a conduit or even as habitat (i.e., a source) (Andren et al. 1997; Dunford and Freemark 2004; Kupfer et al. 2006; Kuefler 2010; Aben et al. 2012). This confounding of patch isolation/connectivity due to matrix quality, although present in most systems (Fahrig 2013; Fletcher et al. 2018), should be more recognizable and addressable as image and landscape component spatial resolution, associated classification accuracy (Aben et al. 2012), and the potential for species group-habitat specificity increase (Hanski 2015).

For analytical purposes, HR GIS imagery fosters identification and delineation of internally more uniform (ecologically relevant) patch types (Fahrig 2013:1650; Rechsteiner et al. 2019).

SUPPLEMENTAL LITERATURE CITED

Aben J, Adriaensen F, Thijs KW, Pellikka P, Siljander M, Lens L, Matthysen E (2012) Effects of matrix composition and configuration on forest bird movements in a fragmented Afromontane biodiversity hot spot. Anim Conserv 15:658–668.

Andrén H, Delin A, Seiler A (1997) Population response to landscape changes depends on specialization to different landscape elements. Oikos 80:193–196

Betts, MG, Fahrig L, Hadley AS, Halstead KE, Bowman J, Robinson WD, Wiens JA, Lindenmayer DB (2014) A species centered approach for uncovering generalities in organism responses to habitat loss and fragmentation. Ecography 37:517-527

Bertin, RI (1977) Breeding habitats of the wood thrush and veery. Condor 79:303-311.

Bevier L, Poole AF, Moskoff W (2004) Veery (*Catharus fuscescens*). In: Poole A (ed) The birds of North America online. Cornell Lab of Ornithology, Ithaca, NY. http:/bna.birds.cornell.edu/BNA/account/Veery/.doi:10.2173/bna.142

Colwell RK, Winkler DW (1984) A null model for null models in biogeography. In: Strong DA Jr., Simberloff D, Abele LG, Thistle AB (eds) Ecological communities: conceptual issues and the evidence. Princeton University Press, Princeton, pp 344-359

DeGraaf RM, Rudis DD (1986) New England wildlife: habitat, natural history, and distribution. USDA For Serv Gen Tech Rep NE-108, Broomall, PA

Desrochers A, Renaud C, Hochachka W, and Cadman M (2010) Area-sensitivity by forest songbirds: theoretical and practical implications of scale-dependency. Ecography, 33, 921–931

Dickson JG, Conner RN, Williamson JH (1983) Snag retention increases bird use of a clear-cut. J Wildl Manage 47:799-804

Dunford W, Freemark K (2004) Matrix matters: effects of surrounding land uses on forest birds near Ottawa, Canada. Landscape Ecol 20:497-511

Farrell SL, Collier BA, Skow KL, Long AM, Campomizzi AJ, Morrison ML, Hays KB, Wilkins RN (2013) Using LiDAR-derived vegetation metrics for high-resolution species distribution models for conservation planning. Ecosphere 4:42. http://dx.doi.org/10.1890/ES12-000352.1

Gallant AL (2009) What you should know about land-cover data. J Wildl Manage 73:796-805

Holland JD, Bert DG, Fahrig L (2004) Determining the spatial scale of species' response to habitat. Bioscience 54:227-233

Kupfer JA, Malanson GP, Franklin SB (2006) Not seeing the ocean for the islands: the mediating influence of matrix-based processes on forest fragmentation effects. Glob Ecol Biogeogr 15:8-20

Huston MA (2002) Introductory essay: Critical issues for improving predictions. In: Scott JM, Heglund PJ, Morrison ML (eds) Predicting species occurrences: Issues of accuracy and scale. Island Press, Washington DC, pp 7-21

Luoto M, Kuussaari M, Toivonen T (2002) Modeling butterfly distribution based on remote sensing data. J Biogeogr 29:1027-1037

Margules CR, Stein JL (1989) Patterns in the distribution of species and the selection of nature reserves: An example from Eucalyptus forests in South-eastern New South Wales. Biol Conserv 50:219-238

McDermid GJ, Hall RJ, Sanchez-Azofeifa GA, Franklin SE, Stenhouse GB, Kobliuk T, LeDrew EF (2009) Remote sensing and forest inventory for wildlife habitat assessment. For Ecol Manage 257:2262-2269

Hejl SJ, Newlon KR, McFadzen ME, Young, JS and Ghalambor CK (2002) Brown Creeper (*Certhia americana*). In: Poole A, Gill F (eds) The birds of North America, no. 669. The Birds of North America, Inc., Philadelphia, PA

Huston MA (2002) Introductory essay: Critical issues for improving predictions. In: Scott JM, Heglund PJ, Morrison ML (eds) Predicting species occurrences: Issues of accuracy and scale. Island Press, Washington DC, pp 7-21

Luoto M, Kuussaari M, Toivonen T (2002) Modeling butterfly distribution based on remote sensing data. J Biogeogr 29:1027-1037

Margules CR, Stein JL (1989) Patterns in the distribution of species and the selection of nature reserves: An example from Eucalyptus forests in South-eastern New South Wales. Biol Conserv 50:219-238

McDermid GJ, Hall RJ, Sanchez-Azofeifa GA, Franklin SE, Stenhouse GB, Kobliuk T, LeDrew EF (2009) Remote sensing and forest inventory for wildlife habitat assessment. For Ecol Manage 257:2262-2269

Meehan TD, Jetz W, and Brown JH (2004) Energetic determinants of abundance in winter landbird communities. Ecol Lett 7:532–537

O'Connor RJ, Fuller RJ (1983) Bird population responses to habitat. In: Taylor K, Fuller RJ, Lack PC (eds) Bird Census and Atlas Studies. Proc VIII International Conf on Bird Census and Atlas Work. Buckinghamshire, England pp 197-211

Paine RT (1966) Food web complexity and species diversity. Am Nat 100:65-75

Paine, RT (1984) Ecological determination in the competition for space. Ecology 65:1399-1348

Pravosudov VV, Grubb TC Jr (1993) White-breasted Nuthatch (Sitta carolinensis). In: Poole A, Gill F (eds) The Acad Nat Sciences, Philadelphia, PA, and The American Ornithologist's Union, Washington, DC

Rechsteiner C, Zellweger F, Gerber G, Breiner FT, Bollmann K (2017) Remotely sensed forest habitat structures improve regional species conservation. Remote Sens Ecol Conserv 3:247–258

Robinson TR, Sargent RR, Sargent MB (1996) Ruby-throated hummingbird (Archilochus colubris). In: Poole A, Gill F (eds) The birds of North America, no. 669. The Birds of North America, Inc., Philadelphia, PA

Root T (1988) Energy constraints on avian distributions and abundances. Ecology 69:330–339

Schoener TW (1983) Simple models of optimal feeding-territory size: a reconciliation. Am Nat 121:608-629

Scott VE (1979) Bird response to snag removal in ponderosa pine. J For 77:26-28

Shirley SM, Yang Z, Hutchinson RA, Alexander JD, McGarigal K, Betts MG (2013) Species distribution modelling for the people: unclassified landsat TM imagery predicts bird occurrence at fine resolutions. Divers Distrib 19:855–866

Shriner SA, Wilson KR, Flather CH (2006) Reserve networks based on richness hotspots and representation vary with scale. Ecol Appl 16:1660-1673

Stohlgren TJ, Chong GW, Kalkhan MA, Schell LD (1997) Multiscale sampling of plant diversity: effects of minimum mapping unit size. Ecol Appl **7:**1064–1074

Stoms DM (1992) Effects of habitat map generalization in biodiversity assessment. Photogramm Eng Remote Sens 58

Van Horn MA, Donovan TM (1994) Ovenbird (*Seiurus aurocapillus*). In: Poole A, Gill F (eds) The birds of North America, no. 88. The Acad Nat Sciences, Philadelphia, PA, and The American Ornithologist's Union, Washington, DC

Wulder MA, Hall RJ, Coops NC, Franklin SE (2004) High spatial resolution remotely sensed data for ecosystem characterization. Biosci 54:511-521

Wiens JA (1977) On competition in variable environments. Am Sci 65:590-597

Yahner RH (1986) Structure, seasonal dynamics, and habitat relationships of avian communities in small even-aged forest stands. Wilson Bull 98:61-82
